# Optimizing cardiovascular disease risk screening in a low-resource setting: cost-effectiveness of program modifications in Sri Lanka modelled with nationally representative survey data

**DOI:** 10.1186/s12889-023-16640-5

**Published:** 2023-09-15

**Authors:** Nilmini Wijemunige, Ravindra P. Rannan-Eliya, Pieter van Baal, Owen O’Donnell

**Affiliations:** 1 Institute for Health Policy, 72 Park Street, Colombo 2, Colombo, Western Province Sri Lanka; 2 Erasmus School of Health Policy & Management, Erasmus University Rotterdam, Rotterdam, The Netherlands; 3 Erasmus School of Economics and Erasmus School of Health Policy & Management, Erasmus University Rotterdam, Rotterdam, The Netherlands

**Keywords:** CVD risk screening, Diabetes, Cost-effectiveness analysis, Modelling study, Low-middle income, Sri Lanka

## Abstract

**Background:**

While screening for cardiovascular disease (CVD) risk can help low-resource health systems deliver low-cost, effective prevention, evidence is needed to adapt international screening guidelines for maximal impact in local settings. We aimed to establish how the cost-effectiveness of CVD risk screening in Sri Lanka varies with who is screened, how risk is assessed, and what thresholds are used for prescription of medicines.

**Methods:**

We used data for people aged 35 years and over from a 2018/19 nationally representative survey in Sri Lanka. We modelled the costs and quality adjusted life years (QALYs) for 128 screening program scenarios distinguished by a) age group screened, b) risk tool used, c) definition of high CVD risk, d) blood pressure threshold for treatment of high-risks, and e) prescription of statins to all diabetics. We used the current program as the base case. We used a Markov model of a one-year screening program with a lifetime horizon and a public health system perspective.

**Results:**

Scenarios that included the WHO-2019 office-based risk tool dominated most others. Switching to this tool and raising the age threshold for screening from 35 to 40 years gave an incremental cost-effectiveness ratio (ICER) of $113/QALY. Lowering the CVD high-risk threshold from 20 to 10% and prescribing antihypertensives at a lower threshold to diabetics and people at high risk of CVD gave an ICER of $1,159/QALY. The findings were sensitive to allowing for disutility of daily medication.

**Conclusions:**

In Sri Lanka, CVD risk screening scenarios that used the WHO-2019 office-based risk tool, screened people above the age of 40, and lowered risk and blood pressure thresholds would likely be cost-effective, generating an additional QALY at less than half a GDP per capita.

**Supplementary Information:**

The online version contains supplementary material available at 10.1186/s12889-023-16640-5.

## Background

Identification of people at high risk for cardiovascular disease (CVD) and managing them with a combination of lifestyle advice and pharmacological treatment is a cornerstone of the World Health Organization (WHO) Package of Essential Noncommunicable Disease Interventions (PEN) [1, 2]. Using a total CVD risk approach, the PEN, supplemented by WHO HEARTS [3, 4], set out guidelines for screening and management of risk factors – hypertension, diabetes, and hypercholesterolaemia – in primary care settings in low- and middle-income countries (LMICs) to prevent CVD.

There is some evidence of improvement in clinical outcomes following PEN implementation [5] and on the cost-effectiveness of PEN variations in South Asia [6]. However, a systematic review of CVD screening programs in LMICs emphasised the importance of assessing the appropriateness of international guidelines in local settings [7]. Designing the pharmacological component of a CVD risk screening and treatment program involves specification of 1) the age groups to screen, 2) the CVD risk prediction tool to use, 3) the threshold to use to identify high CVD risk, 4) whether to lower the blood pressure treatment threshold for people with diabetes, and 5) whether to give statins to all people with diabetes regardless of CVD risk. These choices potentially have important consequences for cost and effectiveness [7–9].

Screening the working-age population can potentially detect CVD risks sufficiently early to avert negative outcomes. But with limited resources, screening at younger ages can have high opportunity costs and may weaken program effectiveness [7].

Most LMICs lack a CVD risk prediction tool derived from domestic data and must rely on tools derived from cohort data from another country [10] or from multiple countries [11]. Validation for use in LMICs is difficult given the lack of accurate morbidity data and longitudinal datasets [12]. The choice of tool may be largely determined by ease of use and WHO endorsement [13]. Compared with an office-based tool, a laboratory-based tool, which requires a blood test for total cholesterol or a lipid profile, is more accurate but also more expensive.

CVD screening programs differ in the CVD risk threshold that is used to trigger statin treatment and, in some programs, to lower the blood pressure threshold for prescription of antihypertensives and determine follow-up frequency. Some countries use lower thresholds of 7.5%—10% risk of a CVD event within ten years [14–16], while others use 20% and above [17, 18]. Many LMICs adopt thresholds used in high-income countries despite possibly facing very different costs and impacts, or they adopt a suggested higher threshold assuming lower affordability [19] but without fully considering lower prices at which medicines may be available.

For prescription of antihypertensives, recent WHO guidelines recommend lower blood pressure thresholds for diabetics (≥ 130/80 mmHg) [3, 4], and for people at high risk of CVD (≥ 130 mmHg) [20]. Several guidelines recommend statins for diabetics without consideration of CVD risk [3, 21, 22].

Since 2011, Sri Lanka has set up over 1,000 Healthy Lifestyle Centres (HLCs) to detect people with high CVD risk and associated risk factors [23, 24]. CVD risk screening and treatment guidelines were published in 2012 [25]. In 2018, these were updated by reducing the CVD risk threshold from 30 to 20% for prescription of statins, and broadening the age group screened from 40–65 years to 35 years and above [17]. There was no published analysis of the health and cost consequences of these changes. Nor has there been analysis of the cost-effectiveness of the current program compared with alternatives that would screen at 40 years and above, use an alternative CVD risk tool, lower the CVD risk threshold to 10% in line with several high-income countries, lower the blood pressure treatment threshold for diabetics and those with high CVD risk, and prescribe statins for all diabetics. By conducting such cost-effectiveness analysis, this study aimed to help decision makers in Sri Lanka, and possibly elsewhere, optimise the CVD risk screening program.

## Methods

### Data

We used data from the Sri Lanka Health and Ageing Study (SLHAS), which is a nationally-representative sample of 6,665 adults aged 18 years and older interviewed in 2018/9. The sample was selected using stratified, multi-stage cluster random sampling [26]. Weights were applied to make the sample representative of the adult population of Sri Lanka in 2019. The dataset had risk factor data needed to simulate screening and predict CVD events for each individual [27]. Except for smoking, each predictor was missing at random in < 1% of the sample. Smoking status was missing for 2.9% of all participants, 3.8% of females, and 4.1% of urban participants. We used chained multiple imputation to impute missing data.

### Screening scenarios

We simulated screening of a cohort of adults aged 35 years and older with no previous history of CVD (angina, coronary artery disease, myocardial infarction or stroke). We modelled programs that would screen at 70% of the capacity of HLCs (20 patients per week in 1,000 HLCs) for 48 weeks of one year (672,000 people), with follow-up of the cohort for 10-years.

**Fig. 1:**
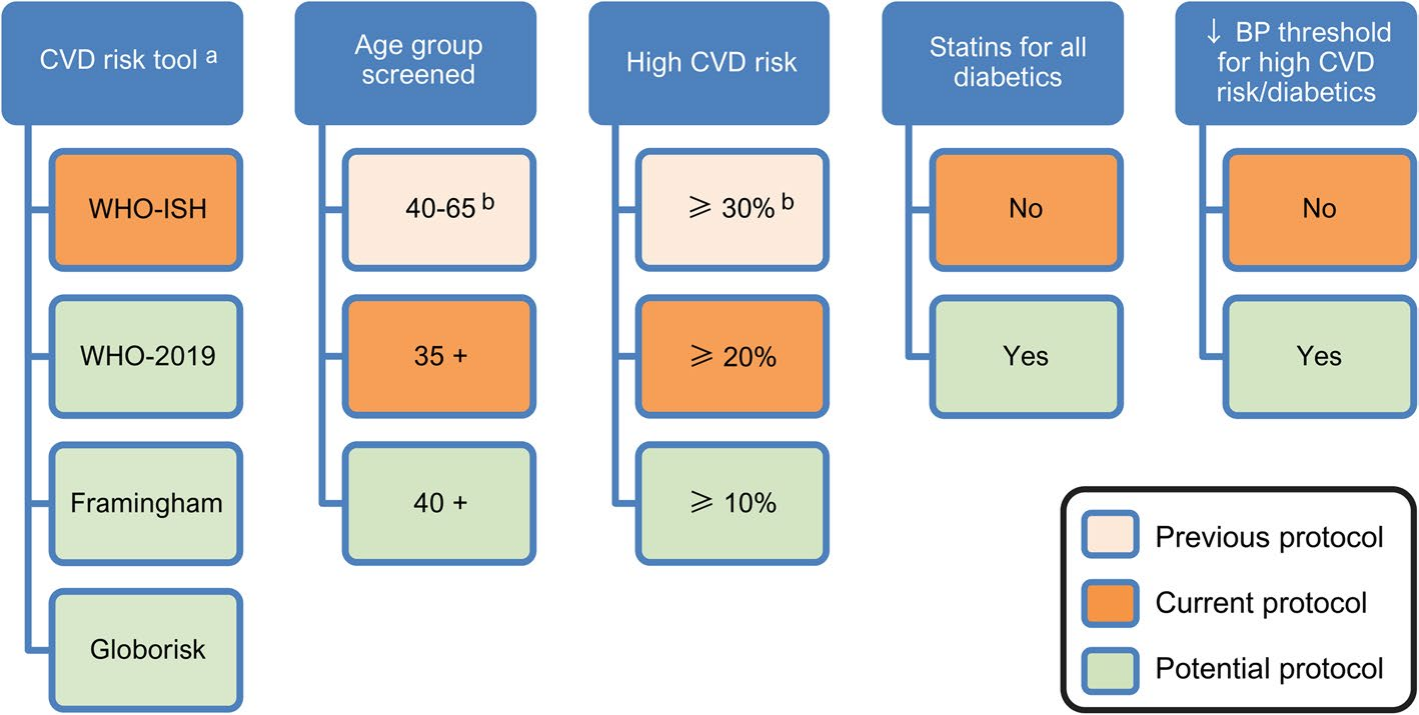
Screening and treatment parameters modelled in previous protocol, current protocol and potential scenarios. *Notes*: ^a^ Office- and lab-based risk tools modelled. Current protocol deviates from previous protocol only in age group screened and definition of high CVD risk. ^b^ These parameter values were only assessed as part of the previous protocol. In all, there were 129 scenarios (8 × 2 × 2 × 2 × 2 + 1) including the base case. In the main text, we show results for scenarios that used WHO-ISH and WHO-2019 tools. Results for scenarios that used Framingham and Globorisk are in Additional file 1

We modelled two main modifications to the current screening protocol that would change the risk prediction tool and the age group screened (Fig. 1). We compared eight screening tools consisting of office- and laboratory-based versions of each of WHO International Society of Hypertension (WHO-ISH) [11], WHO-2019 [28], Framingham [10], and Globorisk [29] (current protocol: office-based WHO-ISH). We used tool-specific definitions of each risk factor used for predictions (Additional file 1: Table S1). We compared three age groups: 40–65 years (previous protocol), 35 years and older (35 +) (current protocol), and 40 years and older (40 +). In all scenarios modelled, screening included glucose tests. Scenarios that used lab-based risk tools also included cholesterol tests in the initial screening.

### Treatment scenarios

In all scenarios modelled, those with blood pressure ≥ 140/90 would be given antihypertensives. Those with fasting blood glucose ≥ 126 mg/dL or random blood glucose ≥ 200 mg/dL would be given hypoglycaemics. In scenarios with lab-based risk tools, those with a total cholesterol ≥ 300 mg/dL would be given statins.

We modelled scenarios that differed in the criteria used to treat additional groups with statins and antihypertensives. First, we varied the CVD high-risk threshold that is used to determine eligibility for statins, and for lowering the blood pressure threshold in some scenarios, from 30% (previous protocol) to 20% (current protocol) to 10% (potential protocol). Second, we modelled giving statins to all diabetics irrespective of total cholesterol and CVD risk. Third, we modelled lowering the blood pressure threshold for prescription of antihypertensives to 130/80 for all diabetics and those classified as high CVD risk.

We modelled follow-up according to the Sri Lankan screening guidelines [17]. Anyone classified as high CVD risk was assumed to be followed up and given a glucose test twice per year. Those who were not high CVD risk but who qualified for any medication were assumed to be followed up and given a glucose test once per year, with an additional follow-up in the initial year. We assumed that all those qualifying for these medications continued to require them after the first year of follow-up. For scenarios with laboratory-based risk tools, total cholesterol was assumed to be measured on each follow-up visit if the initial CVD risk was high. For diabetics, we only modelled follow-up for management of CVD risk, not for diabetes management.

### Outcomes

**Fig. 2:**
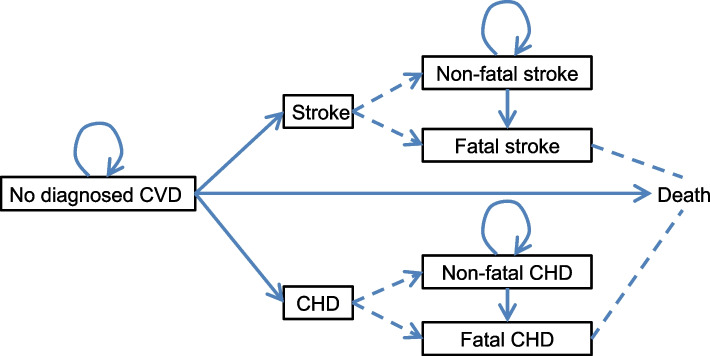
Markov model of population with no known history of CVD. *Notes:* All participants started with no diagnosed CVD. Each solid arrow shows a possible transition at each cycle. Dashed lines with arrow show the breakdown within the same cycle: *e.g.*, a stroke event is either non-fatal or fatal, and fatal stroke is related to death

We used a Markov model with a 1-year cycle for the first 10 years of modelling (Fig. 2). For all scenarios, we fed each individual’s risk factor data into the WHO-2019 laboratory risk tool to estimate the 10-year probability of developing each of coronary heart disease (CHD) and stroke assuming that this tool would be the most accurate for the Sri Lankan population (Additional file 1: Figure S1). We converted the 10-year probabilities to 1-year probabilities (Additional file 1: Text S2). The WHO-2019 tool defined CHD as International Classification of Disease-10 (ICD-10) code I21-I25, and stroke as I60-I69. We utilised 2019 Global Burden of Disease estimates of deaths and incidence of ischaemic heart disease and stroke, by sex and five-year age group [30] to produce mortality ratios for each individual age (Additional file 1: Figure S2). The transition probabilities for a non-fatal event to death in subsequent cycles were obtained by transforming 5-year mortality rates post CHD from 1990–1999 in the Framingham cohort [31] and post stroke from 2000–2004 in a Singaporean cohort study [32]. The transition probability from no diagnosed CVD, to death without any CVD event was calculated by age and sex using WHO life tables [33]. Since the focus is on primary prevention, we did not model multiple CVD events, which would be influenced by the intensity of secondary prevention [34]. Mortality risks from non-fatal events included elevated mortality risk caused by any subsequent CVD event. We assumed that all people alive at the end of 10 years will transition to death using the probability of natural death for that age group, regardless of whether they had a CVD event or not. Cycles continued for each participant until death or the participant reached 100 years of age. Each transition was half-cycle corrected.

The baseline utility at the start of year 1 was calculated for each participant using a Sri Lankan valuation of their responses to the EQ-5D-5L questionnaire [35]. For each subsequent yearly cycle, we calculated the utility for each individual by applying the marginal disutility of one year increase in age, as well as non-fatal stroke and non-fatal CHD for people who transitioned to these states (Table 1).

**Table 1:** Input and sensitivity parameters

Parameter	Value (95% CI)	Distribution	Source
**Events**			
10-year probability of CHD / stroke event	Risk factor specific rates		WHO CVD Risk Chart Working Group [28]
10-year probability of death from CVD event	Age-sex specific proportion applied to CHD events and stroke events	± 10% (uniform)	Global Burden of Disease Collaborative Network [30]
1-year probability of death without previous CVD event	Age-sex specific natural mortality from life-tables		World Health Organization [33]
1-year probability of dying after non-fatal CHD event	0.03 (0.01, 0.04)	limits of 95% CI (β)	Velagaleti, Pencina, Murabito [31]
1-year probability of dying after non-fatal stroke event	0.10	± 20% (uniform)	Sun, Lee, Heng [32]
**Statin treatment**			
Cost for one year of treatment of atorvastatin 20 mg per day (USD)	3.98	± 10% (Uniform)	IHP analysis [36]
RR of non-fatal MI	0.74 (0.67, 0.81)	95% CI (log normal)	Mills, Wu, Chong [37]
RR of non-fatal stroke	0.86 (0.78, 0.95)	95% CI (log normal)	Mills, Wu, Chong [37]
RR of fatal MI	0.82 (0.75, 0.91)	95% CI (log normal)	Mills, Wu, Chong [37]
RR of fatal stroke	0.92 (0.80, 1.07)	95% CI (log normal)	Mills, Wu, Chong [37]
**Antihypertensive treatment**			
Cost for one year of treatment of enalapril 5 mg per day (USD)	2.21	Gamma distribution, assuming 10% standard deviation	IHP analysis [36]
Cost for one year of treatment of nifedipine SR 20 mg per day (USD)	0.78	Gamma distribution, assuming 10% standard deviation	IHP analysis [36]
RR of non-fatal MI	0.86 (0.76, 0.96)	95% CI (log normal)	Brunstrom and Carlberg [38]
RR of non-fatal stroke	0.86 (0.72, 1.01)	95% CI (log normal)	Brunstrom and Carlberg [38]
RR of fatal MI	0.86 (0.65, 1.14)	95% CI (log normal)	Brunstrom and Carlberg [38]
RR of fatal stroke	0.86 (0.65, 1.14)	95% CI (log normal)	Brunstrom and Carlberg [38]
**Anti-diabetic treatment**			
Cost of one year of treatment with metformin 500 mg three times a day (USD)	6.27	Gamma distribution, assuming 10% standard deviation	IHP analysis [36]
**Screening costs (USD)**			
Glucose test	0.17	Gamma distribution, assuming 10% standard deviation	IHP analysis [36]
Total cholesterol test	0.19	Gamma distribution, assuming 10% standard deviation	IHP analysis [36]
Consultation	1.96	Gamma distribution, assuming 10% standard deviation	Amarasinghe, Dalpatadu and Rannan-Eliya [39], Ministry of Health [24]
**Adjustment of annual usual care costs**			
Inflation of usual inpatient and outpatient care costs for general public	1.00	± 20% (uniform)	Authors’ analysis (Additional file 1: Text S1)
Inflation of usual inpatient care costs for people with CHD	2.85 (1.79, 4.54)	95% CI (log normal)	Authors’ analysis (Additional file 1: Text S1)
Inflation of usual inpatient care costs for people with stroke	1.09 (0.53, 2.26)	95% CI (log normal)	Authors’ analysis (Additional file 1: Text S1)
Inflation of usual outpatient care costs for people with CHD	1.95 (1.45, 2.61)	95% CI (log normal)	Authors’ analysis (Additional file 1: Text S1)
Inflation of usual outpatient care costs for people with stroke	1.97 (0.83, 4.69)	95% CI (log normal)	Authors’ analysis (Additional file 1: Text S1)
**Event costs (USD)**			
Cost of myocardial infarction admission	318	± 10% (uniform)	Perera, Rannan-Eliya, Senanayake [40], Amarasinghe, Dalpatadu and Rannan-Eliya [39]
Cost of stroke admission	241	± 10% (uniform)	Perera, Rannan-Eliya, Senanayake [40], Amarasinghe, Dalpatadu and Rannan-Eliya [39]
**Disutilities**			
Non-fatal MI	-0.0210 (-0.066, 0.024)	95% CI (log normal)	Wijemunige, Gamage, Rannan-Eliya (Wijemunige et al.: Population norms and disutility catalogue for chronic conditions in Sri Lanka, forthcoming)
Non-fatal stroke	-0.2493 (-0.340, -0.158)	95% CI (log normal)	Wijemunige, Gamage, Rannan-Eliya (Wijemunige et al.: Population norms and disutility catalogue for chronic conditions in Sri Lanka, forthcoming)
1 year increase in age	-0.0066 (-0.007, -0.006)	95% CI (log normal)	Wijemunige, Gamage, Rannan-Eliya (Wijemunige et al.: Population norms and disutility catalogue for chronic conditions in Sri Lanka, forthcoming)

A log normal distribution was used for disutility as the confidence interval for non-fatal MI spanned positive and negative values

### Impact of treatment

We used estimates from a metanalysis [37] for the effects of statins on the probabilities of non-fatal stroke and myocardial infarction (as a proxy for CHD). We used another metanalysis [38] for the effects of antihypertensives on the risks of CHD and stroke. We used the conservative estimates of these effects for a baseline blood pressure of 140–159 mmHg. Since this meta-analysis did not distinguish between effects of antihypertensives on CHD and stroke mortality, we used the estimated effect on cardiovascular disease mortality for both conditions. We also assumed, conservatively, that there would be no reduction of CHD or stroke risk after treatment of 10 years, though treatment would continue for the individual’s lifetime.

### Costs

We calculated costs over a lifetime horizon from a public health system perspective. Medicines costs, laboratory costs, admission costs for CHD and stroke, and costs of usual care for 2019 were calculated using locally available data (Additional file 1: Text S1). All costs were converted to December 2019 US dollars (US$1 = LKR 181.63), which is the time the SLHAS was completed, the year for which most cost data were available, and an effective way to handle costing in a setting with fluctuating inflation [41].

### Cost-effectiveness analysis

The base-case scenario was the current Sri Lanka CVD screening program (Fig. 1). An incremental cost-effectiveness ratio (ICER) was calculated for each alternative scenario. Incremental costs were plotted against incremental QALYs, and cost-effectiveness frontiers drawn. We identified scenarios that were strongly dominated (another scenario produced more QALYs at lower cost) or weakly dominated (another scenario produced more total QALYs at a lower ICER). As there were no cost-effectiveness thresholds (CET) derived from local data, we compared ICERs to a threshold of gross domestic product (GDP) per capita [42]. We also used lower thresholds of half and a quarter of a GDP per capita based on application of an approach to estimate CETs [43–45]. We used 2019 GDP per capita in current US dollars of $4,083 [46]. All costs and QALYs were discounted at 3% per year [7]. Subgroup analysis of costs and impact was performed by 5-year age-groups for selected scenarios.

### Sensitivity analysis

In a deterministic sensitivity analysis, we tested sensitivity to increasing the effect of antihypertensives on the risk of non-fatal stroke to the higher estimate at SBP ≥ 160 mmHg [33]. We tested reducing the discount rate to 0% and raising it to 6%. We tested the effect of lowering the utility score for myocardial infarction to a value similar to that of stroke [47, 48]. We also tested changing the cost of usual care to 80% and 120% of the value used, and changed the ratio used to inflate the cost of usual care for all people with CHD and stroke to 1 and 3.

In a separate one-way sensitivity analysis, we applied a disutility of 0.00384 to all participants newly prescribed medication, based on a small study from a high-income country [49] to account for a possible burden of taking long-term daily medications [49–51]. Although this value aimed to capture the inconvenience of taking medications, “pill disutility” could also apply to the possibility of side-effects [49].

Probabilistic sensitivity analysis (PSA) was performed on all scenarios on the cost-effectiveness frontier that used WHO-ISH or WHO 2019 risk tools, which are most likely to be considered by the Sri Lankan Ministry of Health (MOH). 1,000 simulations were performed, randomly drawing from the distributions in Table 1, and cost-effectiveness acceptability curves (CEAC) were plotted.

We reported using the Consolidated Health Economic Evaluation Reporting Standards (CHEERS) checklist [52] (Additional file 1: Table S2). All analyses were done using Stata V.17.0 (Stata, College Station, Texas, USA).

## Results

**Fig. 3:**
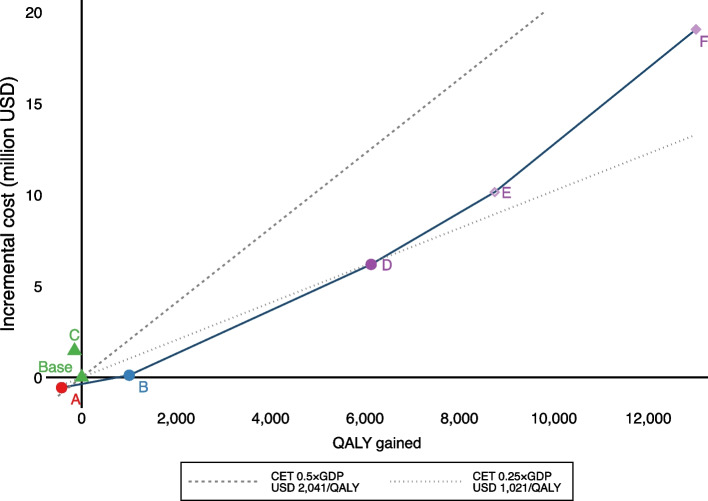
Cost-effectiveness frontier for all scenarios using WHO-ISH and WHO-2019 risk tools. *Notes*: Letters denote scenarios labelled in Table 2. Triangles denote scenarios that used the WHO-ISH office tool, filled circles denote scenarios that used the WHO-2019 office tool. The hollow diamond denotes a scenario that used the WHO-2019 tool with hypertension medication at a lower blood pressure threshold. The full diamond denotes a scenario that used the WHO-2019 tool with statins for all diabetics and hypertension medication at a lower blood pressure

Table 2 shows the percentage of people newly prescribed medications, the incremental QALYs, incremental costs and ICERs compared to the base case, for selected scenarios that used the WHO-ISH or WHO-2019 risk tools. The same scenarios are shown along with the cost-effectiveness frontier in Fig. 3. The base case, which modelled the current screening protocol (WHO-ISH office tool, 20% risk threshold, 35 +), the old protocol (WHO-ISH office tool, 30% risk threshold, 40–65) and all non-dominated scenarios are included. The results for all other scenarios, including those that used the Globorisk and Framingham screening tools, are shown in Additional file 1: Table S3 and Table S4. Table 2 also shows the ICER from the nearest scenario on the cost-effectiveness frontier.

**Table 2:** Incremental costs, QALYs and ICERs of selected scenarios

Scenario	CVD risk tool	High CVD risk	Ages screened	Statins all diabetics	HTN medication at lower BP	% of screened people newly commenced on:				Incre-mental costs (million $)	Incre-mental QALYs	ICER ($/QALY)	ICER from previous scenario on CEF^a^ ($/QALY)
						Anti-hypertensive	Statin	Anti-diabetic	At least 1 medication				
Base	WHO-ISH, office	≥ 20%	35 +	NO	NO	19.8	2.4	8.1	21.2	[Base]	[Base]	[Base]	[Base]
A	WHO 2019, office	≥ 20%	35 +	NO	NO	19.8	2.1	8.1	21.4	-0.6	-426	Cost saving	-
B	WHO 2019, office	≥ 20%	40 +	NO	NO	21.7	2.7	8.5	22.5	0.1	1,007	113	113
C	WHO-ISH, office	≥ 30%	40–65	NO	NO	21.2	0.9	8.8	22.3	1.5	-153	Dominated	-
D	WHO 2019, office	≥ 10%	40 +	NO	NO	21.7	14.5	8.5	26.1	6.2	6,129	1,009	1,185
E	WHO 2019, office	≥ 10%	40 +	NO	YES	30.5	14.5	8.5	27.2	10.1	8,747	1,159	1,511
F	WHO 2019, office	≥ 10%	40 +	YES	YES	30.5	31.1	8.5	28.5	19.0	13,010	1,464	2,090

Scenario labels as used in Fig. 3. CEF = cost-effectiveness frontier. ^a^ ICERs are calculated from the closest least costly scenario on the CEF. For example, moving from scenario D to scenario E costs $1,511/QALY ($10.1 million—$6.2 million) / (8,747 QALYs – 6,129 QALYs)

Of the 672,000 people screened, the percentage of people newly commenced on at least one of the three medications ranged from 21.2% – 30.5% for antihypertensives, 0.9% – 14.5% for statins, 8.1% – 8.5% for antidiabetics, and 22.3% to 27.2%% for at least one of the three medications. The incremental cost ($19.0 million) of the most expensive scenario on the cost-effectiveness frontier, for screening one cohort and following this cohort over a lifetime is estimated to be 1.5% of the government’s annual recurrent health expenditure in 2019 ($1.3 billion).

The old protocol (Scenario C) was dominated by the current protocol (Scenario “Base” in Table 2). Switching from the WHO-ISH to WHO-2019 tool (Scenario A) was cost saving, with a small loss in QALYs. Using the WHO-2019 tool and changing the age group screened to 40 + (Scenario B) had an ICER of $113 compared to the base case. Reducing the risk threshold to 10% (Scenario D) resulted in a gain of 6,129 QALYs with an ICER of $1,009/QALY. Moving from one scenario to the next most effective scenario along the frontier cost around 0.25 × to 0.5 × GDP per capita per QALY).

### WHO-2019 lab tool with cholesterol testing compared to WHO-2019 office tool

The incremental costs and QALYs gained from using the WHO-2019 lab tool are compared to the office tool in Additional file 1: Figure S3 for four combinations of ages screened (35 + or 40 +) and risk thresholds (10% or 20%). Compared with the base case, the ICER of most scenarios using the lab tool are below 0.5 × GDP per capita per QALY. All are dominated by scenarios using the WHO-2019 office tool.

### Statins for all diabetics and lowering BP threshold for high-risk individuals

The impact of adding statins for diabetics (SD) and a lowered blood pressure threshold (LBP) for high-risk individuals is shown in Fig. 3 (Scenarios E and F) and Additional file 1: Figure S4. Compared to the base case, adding SD and LBP to the scenarios using WHO-2019 office tools had an ICER of approximately 0.5 × GDP per capita/QALY or less. However, most of the scenarios with SD or LBP lie above the cost-effectiveness frontier (meaning they are dominated by other more cost-effective alternatives). There are two exceptions: Scenario E, which added LBP to scenario D (WHO-2019, 10%, 40 +) has an ICER of 1,511 (less than 0.5 × GDP per capita/QALY) compared to scenario D, and lies on the cost-effectiveness frontier. Scenario F, which further adds SD to scenario E, also lies on the cost-effectiveness frontier, however it has a higher ICER of $2,090/QALY (0.5 × GDP per capita/QALY) compared to scenario E.

### Framingham and Globorisk tools

The impact of switching from WHO-2019 office to Framingham office and Globorisk office tools are shown in Additional file 1: Figure S5. Almost all scenarios with Framingham and Globorisk tools are dominated by scenarios using WHO-2019 office tools. Globorisk scenario G, which uses a 20% threshold, lies on the cost-effectiveness frontier close to scenarios using the WHO-2019 tool with a 10% threshold. Whilst some scenarios on the cost-effectiveness frontier using the Globorisk tool generate the highest impact, they also have larger ICERs (0.8 – 2.3 × GDP per capita/QALY) in comparison to the closest cheaper model on the frontier.

### Sensitivity analysis

**Fig. 4:**
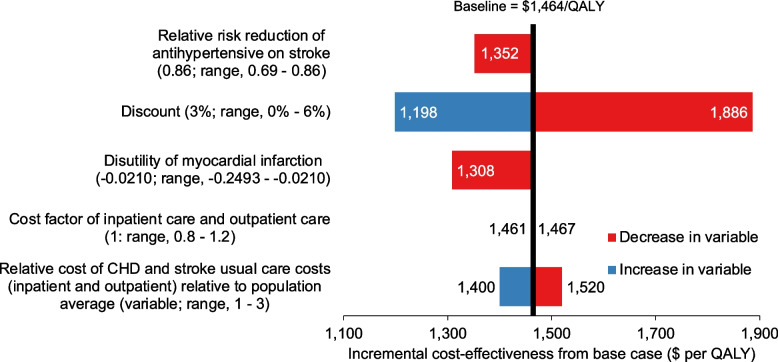
One-way sensitivity analysis assessing cost-effectiveness of Scenario F (WHO-2019 office, 10%, 40 + , SD, LBP). *Notes:* SD = statins for diabetics, LBP = lowered blood pressure threshold

Results of the deterministic sensitivity analysis are shown in Fig. 4 for Scenario F (WHO-2019 office, 10% threshold, 40 + , SD and LBP). Increasing the impact of antihypertensives on stroke reduced the ICER by 8% to $1,352/QALY from $1,464/QALY. Increasing the discount rate to 6% reduced the ICER to $1,198/QALY, whilst removing the discount rate increased the ICER to $1,886/QALY. Increasing the disutility of having a myocardial infarction reduced the ICER to $1,308/QALY. Deflating and inflating the estimated costs for usual care by 20% changed the ICER marginally. Reducing the relative costs of usual care for CHD stroke patients to be the same as the general population increased the ICER to $1,520/QALY, and increasing the relative costs to be three times that of the general population reduced the ICER to $1,400 per QALY. Results from the PSA are shown in Additional file 1: Figure S6. All scenarios on the cost-effectiveness frontier have more than an 90% probability of having an ICER of 0.5 × GDP per capita/QALY or less.

### Sensitivity to pill-taking disutility

Overall, when pill-taking disutility is included, the QALY gain is diminished and ICERs increase, particularly in scenarios which place a large proportion of individuals on new medication. Scenarios using the Globorisk tool (H, I) on the cost-effectiveness frontier, and scenarios using the Framingham and Globorisk tool with 10% thresholds move from costing less than 0.5 × GDP per capita/QALY when the pill-taking disutility is set to 0 (Additional file 1: Figure S5), to more than 0.5 × GDP per capita/QALY when pill-taking disutility is -0.00384 (Additional file 1: Figure S7).

### Impact by age group

The incremental costs and impact by age group for Scenario F (WHO-2019 office, 10% threshold, 40 + , SD and LBP scenario) are shown in Additional file 1: Figure S8. In general, older age groups have lower ICERs than younger age groups.

## Discussion

Switching the Sri Lankan CVD screening program from the WHO-ISH to WHO-2019 office risk tool would be cost-effective and have a far higher impact, particularly if combined with lowering the high-risk threshold to 10%. Raising the lower age threshold for screening from 35 to 40 years has a very low ICER of $113/QALY. Although lifetime exposure to low-density lipoprotein cholesterol, including in early adulthood can pose a great CVD risk, and so interventions should not neglect younger people [53], we recognise that the health system has limited screening capacity and resources. Given these constraints, greater impact and better cost-effectiveness are achieved by screening adults aged 40 and above. The ICER of younger age-groups is consistently higher than older age groups (Additional file 1: Figure S8).

Prescribing antihypertensives at a lower threshold to people classified as high-risk of CVD and diabetics has an ICER of around 0.25 × GDP per capita/QALY. Prescribing statins to all diabetics regardless of CVD risk is somewhat more expensive, with some scenarios costing around 0.5 × GDP per capita/QALY. Age and gender-specific risk thresholds may be needed for statin initiation given possible side effects, which increase with age [19]. However, a recent metanalysis did not find an increased risk in serious adverse events with low-intensity statin treatment [54]. Nevertheless, in sensitivity analyses, when we modelled a disutility for taking medications, which could also include disutility from potential side-effects, ICERs for all scenarios increased. The scenarios using the WHO-2019 office tool on the cost-effectiveness frontier were robust, and remained on or very close to the new cost-effectiveness frontier, and still had ICERs less than 0.5 × GDP per capita/ QALY. However, scenarios which resulted in larger proportions of people newly commenced on medications were more sensitive to “pill disutility”.

Compared to the current screening protocol, using the WHO-2019 laboratory tool and including cholesterol testing costs less than 1 × GDP per capita/ QALY. However, as it is dominated by scenarios that use the WHO-2019 office tool, it could be argued that the WHO-2019 office tool may be sufficient in resource-constrained environments.

The study used a public health system perspective, which is appropriate since CVD screening protocols are established by the MOH and public healthcare costs are of most interest to decision makers. A societal perspective would include travel costs to facilities, but also the likely much larger increase in labour productivity from reduced CVD events [55] that would be partially offset by future non-medical expenditures [56]. A lifetime perspective is used as CVD preventative treatment is long-term, and restricting analysis to a shorter time period would not capture long-term costs and benefits [57, 58].

While our modelling suggests that modifications to CVD risk screening in Sri Lanka would be highly cost effective, the efficiency gain may not materialise in a real-world situation [5, 59]. Though limited in size and follow-up duration, some studies in LMICs suggest the impact of PEN interventions can be muted due to inadequate follow-up, high drug costs and poor adherence [60]. Sri Lanka has very low drug costs, as well as an established system for follow-up, although public sector drug availability is facing pressures due to the economic crisis [61] that further underlines the importance of cost-effective preventative medicine [62].

Individual-level risk-factor data used in the model are collected in WHO STEPwise approach to surveillance (STEPS) surveys in LMICs [63], suggesting that similar analyses may be feasible in other LMICs with CVD screening programs.

Our findings may assist healthcare policy makers in Sri Lanka to further refine the CVD risk screening protocol for maximal impact. It enriches the evidence base to guide policy makers elsewhere in designing screening protocols that implement the PEN and HEARTS packages.

### Limitations

As Sri Lanka does not have data to accurately estimate willingness to pay for QALYs, we could not define cost-effectiveness thresholds that could be used to identify program scenarios as highly, moderately, or not cost-effective. Instead, we provided incremental cost per QALY for all scenarios on the cost-effectiveness frontier [42]. We also compared the ICERs with thresholds of 0.25, 0.5 and 1 × GDP per capita/QALY [64].

We did not recalibrate the CVD risk prediction tools for use in Sri Lanka given the lack of high-quality data on incidence of CVD in the country. However, we do not aim to establish whether each risk tool is accurate, and we did model scenarios that set high CVD risk at various thresholds for each tool.

Several limitations could lead to upwardly biased estimates of ICERs. First, we assumed no impact on morbidity and mortality beyond 10 years, although we modelled the costs of treatment and follow-up over a lifetime. It is possible that the reduction in CVD risks persist beyond 10 years. Second, since the focus was on cardiovascular disease, health gains from diabetes screening and management due to reduced microvascular complications, such as diabetic retinopathy, nephropathy and neuropathy, were not modelled, although the costs of hypoglycemics and yearly glucose checks were included. However, it is expected that most of the reduction in disease burden from diabetes and CVD risk screening programs will come from the prevention of cardiovascular disease, rather than the prevention of other complications of diabetes [65]. Third, conservative estimates were used for impacts of treatment with antihypertensives and statins and for the disutility of myocardial infarction. The sensitivity analysis suggests less conservative estimates would reduce the ICER by 18%. Fourth, we allowed for the potential disutility of pill taking in recognition of the possibility that when potentially a substantial number of people are put on medications many may experience side effects or simply resent the effort of routinely taking medicines. However, there is very little research available in LMICs to quantify this disutility, and we relied on an estimate from a small study in a HIC setting.

## Conclusions

Subject to the acknowledged limitations, this study has delivered evidence that modifications to the CVD risk screening program in Sri Lanka would be cost effective. Changing to the WHO-2019 office screening tool, increasing the age at which screening starts to 40 + , lowering the CVD risk threshold for statin treatment to 10%, lowering the blood pressure threshold of high-risk people for prescription of antihypertensives, and prescribing statins to diabetics, are all likely to generate health improvements at reasonable incremental costs.

### Supplementary Information

**Additional file 1: Table S1.** Variables and definitions used for screening tools. **Table S2.** CHEERS checklist. **Table S3.** Incremental costs, QALYs and ICERs of all scenarios (sorted by incremental costs). **Table S4.** Incremental costs, QALYs and ICERs of all scenarios (sorted by scenario). **Table S5.** Distribution of inpatient and outpatient encounters, costs, and cost per capita. **Table S6.** Coefficients and 95% confidence intervals of negative binomial regression to assess the impact of CHD and stroke on inpatient and outpatient encounter numbers.** Figure S1.** Comparison of percentage of people who will develop cardiovascular disease between 2019-2028 by age and prediction tool. **Figure S2.** Mortality rates by age and gender, for coronary heart disease and stroke. **Figure S3.** Comparison of ICERs of the current protocol, and proposed scenarios using the WHO-2019 lab tool versus WHO-2019 office tool. **Figure S4.** Cost‑effectiveness frontier - LBP and SD for WHO 2019 office risk tool.**Figure S5.** Comparison of ICERs of the old program, and proposed programs using any risk tool, and modifying risk thresholds and age-groups screened . **Figure S6.** Probabilistic sensitivity analysis of scenarios on the cost‑effectiveness frontier. **Figure S7.** Impact on cost, impact and cost-effectiveness frontier, when including pill disutility . Figure S8. Incremental costs and QALYs by age category for Scenario G (WHO-2019 office, 10% threshold, 40+, SD and LBP) model compared to the base-case. **Text S1. Text S2.**

## Data Availability

The data that support the findings of this study are available from the SLHAS Consortium but restrictions apply to the availability of these data, which were used under license for the current study, and so are not publicly available. The SLHAS Consortium, which has adopted an Open Data policy will provide access to SLHAS Wave 1 data from 2024, on application to the Consortium by interested researchers. The specific data file used for this paper can be obtained from Nilmini Wijemunige upon reasonable request, and with permission of the SLHAS Consortium.

## Abbreviations

CHEERS: Consolidated Health Economic Evaluation Reporting Standards
CVD: Cardiovascular Disease
GDP: Gross Domestic Product
HEARTS: HEARTS Technical Package for Cardiovascular Disease Management in Primary Health Care
HLCs: Healthy Lifestyle Centres
ICD-10: International Classification of Disease-10
ICERs: Incremental Cost-Effectiveness Ratios
ISH: International Society of Hypertension
LBP: Lowered Blood Pressure threshold
LMICs: Low- and Middle-Income Countries
PEN: Package of Essential Noncommunicable Disease Interventions
QALYs: Quality Adjusted Life Years
SD: Statins for Diabetics
SLHAS: Sri Lanka Health and Ageing Study
WHO: World Health Organization
WHO-ISH: World Health Organization International Society of Hypertension
WHO-2019: World Health Organization 2019
