# Supplementary material for: Optimizing cardiovascular disease risk screening in a low-resource setting: cost-effectiveness of program modifications in Sri Lanka modelled with nationally representative survey data
Source: BMC Public Health. 2023 Sep 15;23:1792. doi: 10.1186/s12889-023-16640-5 (PMC10503056; doi:10.1186/s12889-023-16640-5)
Supplement: Supplementary file 1 — Additional file 1: Table S1. Variables and definitions used for screening tools. Table S2. CHEERS checklist. Table S3. Incremental costs, QALYs and ICERs of all scenarios (sorted by incremental costs). Table S4. Incremental costs, QALYs and ICERs of all scenarios (sorted by scenario). Table S5. Distribution of inpatient and outpatient encounters, costs, and cost per capita. Table S6. Coefficients and 95% confidence intervals of negative binomial regression to assess the impact of CHD and stroke on inpatient and outpatient encounter numbers. Figure S1. Comparison of percentage of people who will develop cardiovascular disease between 2019-2028 by age and prediction tool. Figure S2. Mortality rates by age and gender, for coronary heart disease and stroke. Figure S3. Comparison of ICERs of the current protocol, and proposed scenarios using the WHO-2019 lab tool versus WHO-2019 office tool. Figure S4. Cost‑effectiveness frontier - LBP and SD for WHO 2019 office risk tool.Figure S5. Comparison of ICERs of the old program, and proposed programs using any risk tool, and modifying risk thresholds and age-groups screened . Figure S6. Probabilistic sensitivity analysis of scenarios on the cost‑effectiveness frontier. Figure S7. Impact on cost, impact and cost-effectiveness frontier, when including pill disutility . Figure S8. Incremental costs and QALYs by age category for Scenario G (WHO-2019 office, 10% threshold, 40+, SD and LBP) model compared to the base-case. Text S1. Text S2. [file 12889_2023_16640_MOESM1_ESM.pdf]

ADDITIONAL FILE 1

Optimizing cardiovascular disease risk screening in a low-resource setting: cost-effectiveness of program modifications in Sri Lanka modelled with nationally representative survey data

Nilmini Wijemunige, Ravindra P. Rannan-Eliya, Pieter van Baal, Owen O'Donnell

Table S1 Variables and definitions used for screening tools 2

Table S2 CHEERS checklist 3

Table S3 Incremental costs, QALYs and ICERs of all scenarios (sorted by incremental costs) 6

Table S4 Incremental costs, QALYs and ICERs of all scenarios (sorted by scenario) 8

Table S5 Distribution of inpatient and outpatient encounters, costs, and cost per capita 20

Table S6 Coefficients and 95% confidence intervals of negative binomial regression to assess the impact of CHD and stroke on inpatient and outpatient encounter numbers 21

Figure S1 Comparison of percentage of people who will develop cardiovascular disease between 2019-2028 by age and prediction tool 10

Figure S2 Mortality rates by age and gender, for coronary heart disease and stroke 11

Figure S3 Comparison of ICERs of the current protocol, and proposed scenarios using the WHO-2019 lab tool versus WHO-2019 office tool 12

Figure S4 Cost-effectiveness frontier - LBP and SD for WHO 2019 office risk tool 13

Figure S5 Comparison of ICERs of the old program, and proposed programs using any risk tool, and modifying risk thresholds and age-groups screened 14

Figure S6 Probabilistic sensitivity analysis of scenarios on the cost-effectiveness frontier 15

Figure S7 Impact on cost, impact and cost-effectiveness frontier, when including pill disutility 16

Figure S8 Incremental costs and QALYs by age category for Scenario G (WHO-2019 office, 10% threshold, 40+, SD and LBP) model compared to the base-case 17

Text S1 18

Text S2 22

Table S1 Variables and definitions used for screening tools

Risk assessment method	Screening Parameters							
	Age	Sex	Smoking	Diabetes	SBP	SBP treatment	BMI	Total cholesterol	HDL cholesterol
WHO/ISH office			Within past year	OHG/insulin<br>FG ≥ 126 mg/dL<br>2h ≥ 200 mg/dL				be 5 mmol/L for Sri Lanka	
WHO/ISH, lab			Within past year	OHG/insulin<br>FG ≥ 126 mg/dL<br>2h ≥ 200 mg/dL					
WHO-2019, office			Current						
WHO-2019, lab			Current	Known diabetes<br>FG ≥ 126 mg/dL<br>2h ≥ 200 mg/dL					
Framingham office - D'Agostino (2008)			Current	OHG/insulin<br>FG ≥ 126 mg/dL					
Framingham lab - D'Agostino (2008)			Current	OHG/insulin<br>FG ≥ 126 mg/dL					
Globorisk office			Current						
Globorisk lab			Current	OHG/insulin<br>FG ≥ 126 mg/dL<br>Random ≥ 200 mg/dL					

Notes: OHG = oral hypoglycaemics, FG = fasting blood glucose, 2h = 2 hour post prandial or oral glucose tolerance test, Random = random blood glucose. Oral glucose tolerance and HbA1c test results in our cohort were not used for calculating risk by these tools, since its use in the community is limited. For tools that had 2h glucose criteria, random blood glucose results were considered when other criteria were not fulfilled.

Table S2 CHEERS checklist

Topic	No.	Item	Location where item is reported
**Title**			
	1	Identify the study as an economic evaluation and specify the interventions being compared.	Title
**Abstract**			
	2	Provide a structured summary that highlights context, key methods, results, and alternative analyses.	Abstract
**Introduction**			
**Background and objectives**	3	Give the context for the study, the study question, and its practical relevance for decision making in policy or practice.	Context (general): Introduction, Paragraph 5<br>Context (local): Introduction, Paragraph 6<br>*Study question, practical relevance* - Introduction, Paragraph 6
**Methods**			
**Health economic analysis plan**	4	Indicate whether a health economic analysis plan was developed and where available.	Not applicable
**Study population**	5	Describe characteristics of the study population (such as age range, demographics, socioeconomic, or clinical characteristics).	Methods: Data, Paragraph 1
**Setting and location**	6	Provide relevant contextual information that may influence findings.	Methods: Data, Paragraph 1
**Comparators**	7	Describe the interventions or strategies being compared and why chosen.	Methods: Screening scenarios, Paragraph 2<br>Methods: Treatment scenarios, Paragraph 2<br>*Reasons* - Introduction Paragraph 2-6
**Perspective**	8	State the perspective(s) adopted by the study and why chosen.	Costs<br>*Reasons* - Discussion, Paragraph 4
**Time horizon**	9	State the time horizon for the study and why appropriate.	Methods: Impact of treatment<br>Methods: Costs<br>*Reasons* - Discussion, Paragraph 4

Topic	No.	Item	Location where item is reported
**Discount rate**	10	Report the discount rate(s) and reason chosen.	Methods: Cost-effectiveness analysis
**Selection of outcomes**	11	Describe what outcomes were used as the measure(s) of benefit(s) and harm(s).	Methods: Outcomes, Paragraph 1-2
**Measurement of outcomes**	12	Describe how outcomes used to capture benefit(s) and harm(s) were measured.	Methods: Outcomes, Paragraph 1-2
**Valuation of outcomes**	13	Describe the population and methods used to measure and value outcomes.	Methods: Outcomes, Paragraph 1-2
**Measurement and valuation of resources and costs**	14	Describe how costs were valued.	Methods: Costs<br>Additional file 1: Text S1
**Currency, price date, and conversion**	15	Report the dates of the estimated resource quantities and unit costs, plus the currency and year of conversion.	Methods: Costs<br>Methods: Cost-effectiveness analysis
**Rationale and description of model**	16	If modelling is used, describe in detail and why used. Report if the model is publicly available and where it can be accessed.	Methods: Screening scenarios, Treatment scenarios, Outcomes<br>Data availability statement
**Analytics and assumptions**	17	Describe any methods for analysing or statistically transforming data, any extrapolation methods, and approaches for validating any model used.	Methods: Outcomes<br>Additional file 1: Text S1
**Characterising heterogeneity**	18	Describe any methods used for estimating how the results of the study vary for subgroups.	Methods: Cost-effectiveness analysis
**Characterising distributional effects**	19	Describe how impacts are distributed across different individuals or adjustments made to reflect priority populations.	Not reported
**Characterising uncertainty**	20	Describe methods to characterise any sources of uncertainty in the analysis.	Methods: Sensitivity analysis, Paragraph 1-3
**Approach to engagement with patients and others affected by the study**	21	Describe any approaches to engage patients or service recipients, the general public, communities, or stakeholders (such as clinicians or payers) in the design of the study.	Not applicable

Topic	No.	Item	Location where item is reported
**Results**			
**Study parameters**	22	Report all analytic inputs (such as values, ranges, references) including uncertainty or distributional assumptions.	Table 1
**Summary of main results**	23	Report the mean values for the main categories of costs and outcomes of interest and summarise them in the most appropriate overall measure.	Results, Paragraph 2-3<br>Table 2
**Effect of uncertainty**	24	Describe how uncertainty about analytic judgments, inputs, or projections affect findings. Report the effect of choice of discount rate and time horizon, if applicable.	Results: Sensitivity analysis
**Effect of engagement with patients and others affected by the study**	25	Report on any difference patient/service recipient, general public, community, or stakeholder involvement made to the approach or findings of the study	Not applicable
**Discussion**			
**Study findings, limitations, generalisability, and current knowledge**	26	Report key findings, limitations, ethical or equity considerations not captured, and how these could affect patients, policy, or practice.	Discussion, Paragraph 1-3<br>Discussion: Limitations, Paragraph 1-3
**Other relevant information**			
**Source of funding**	27	Describe how the study was funded and any role of the funder in the identification, design, conduct, and reporting of the analysis	Funding statement
**Conflicts of interest**	28	Report authors conflicts of interest according to journal or International Committee of Medical Journal Editors requirements.	Competing interests statement

CHEERS template from Husereau D, Drummond M, Augustovski F, et al. Consolidated Health Economic Evaluation Reporting Standards 2022 (CHEERS 2022) Explanation and Elaboration: A Report of the ISPOR CHEERS II Good Practices Task Force. Value Health 2022;25. doi:10.1016/j.jval.2021.10.008

Table S3 Incremental costs, QALYs and ICERs of all scenarios (sorted by incremental costs)

Scenario	CVD risk tool	High CVD risk	Ages screened	Statins all diabetics	HTN medication at lower BP	% of screened people newly commenced on:				Incremental costs ($)	Incremental QALYs	ICER ($/QALY)	Dominance
						Anti-hypertensive	Statin	Anti-diabetic	At least 1 medication				
Base	WHO-ISH, office	≥20%	35+			19.8	2.4	8.1	21.2	0.0	0		Base
A	WHO 2019, office	≥20%	35+			19.8	2.1	8.1	21.4	-0.6	-426		CS
B	WHO 2019, office	≥20%	40+			21.7	2.7	8.5	22.5	0.1	1,007	113	ND
	WHO-ISH, office	≥20%	40+			21.7	2.8	8.5	22.3	0.7	1,362	481	ND
C	WHO-ISH, office	≥30%	40-65			21.2	0.9	8.8	22.3	1.5	-153	-9,538	SD
	WHO-ISH, office	≥10%	35+			19.8	6.1	8.1	21.3	2.0	1,593	1,274	ED
	WHO-ISH, office	≥10%	40+			21.7	7.3	8.5	22.4	3.0	3,344	910	ED
	WHO 2019, office	≥20%	35+		Y	26.8	2.1	8.1	22.7	3.2	1,429	2,249	SD
	WHO 2019, lab	≥20%	35+			19.8	7.3	8.1	23.7	3.7	1,225	2,985	SD
	WHO-ISH, office	≥20%	35+		Y	26.7	2.4	8.1	22.6	3.8	1,818	2,072	SD
	WHO 2019, office	≥20%	40+		Y	29.1	2.7	8.5	23.9	3.8	3,123	1,225	SD
	WHO-ISH, lab	≥20%	35+			19.8	6.4	8.1	23.5	3.9	1,076	3,649	SD
	WHO 2019, office	≥10%	35+			19.8	11.4	8.1	24.2	4.2	3,708	1,146	ED
	WHO 2019, lab	≥20%	40+			21.7	8.2	8.5	24.6	4.2	2,939	1,430	SD
	WHO-ISH, office	≥20%	40+		Y	28.9	2.8	8.5	23.7	4.3	3,431	1,268	SD
	WHO-ISH, lab	≥20%	40+			21.7	6.7	8.5	24.3	4.3	2,572	1,682	SD
	WHO-ISH, office	≥10%	35+		Y	26.7	6.1	8.1	22.6	5.8	3,401	1,698	SD
	Globorisk, office	≥20%	35+			19.8	13.5	8.1	24.2	5.9	4,722	1,250	ED
D	WHO 2019, office	≥10%	40+			21.7	14.5	8.5	26.1	6.2	6,129	1,009	ND
	WHO-ISH, office	≥10%	40+		Y	28.9	7.3	8.5	23.7	6.7	5,401	1,242	SD
	Framingham, office	≥20%	35+			19.8	13.9	8.1	23.9	7.1	4,628	1,544	SD
	WHO-ISH, lab	≥10%	35+			19.8	11.5	8.1	23.7	7.4	3,068	2,422	SD
	WHO 2019, lab	≥20%	35+		Y	26.7	7.3	8.1	25.0	7.5	3,044	2,457	SD
	WHO-ISH, lab	≥20%	35+		Y	26.7	6.4	8.1	24.8	7.8	2,883	2,694	SD
	WHO 2019, lab	≥20%	40+		Y	29.0	8.2	8.5	25.8	7.9	5,011	1,583	SD
	WHO-ISH, lab	≥20%	40+		Y	28.9	6.7	8.5	25.6	8.1	4,628	1,746	SD
	WHO 2019, office	≥10%	35+		Y	27.9	11.4	8.1	25.4	8.2	5,957	1,380	SD
	Globorisk, office	≥20%	40+			21.7	17.0	8.5	26.1	8.2	7,390	1,112	ED
	WHO-ISH, lab	≥10%	40+			21.7	13.0	8.5	24.6	8.3	5,025	1,647	SD
	Framingham, office	≥20%	40+			21.7	17.7	8.5	25.8	9.9	7,433	1,332	ED
	WHO 2019, office	≥20%	35+	Y		19.8	20.6	8.1	24.1	10.0	4,646	2,161	SD
	Globorisk, office	≥20%	35+		Y	28.3	13.5	8.1	25.3	10.0	7,113	1,400	SD
E	WHO 2019, office	≥10%	40+		Y	30.5	14.5	8.5	27.2	10.1	8,747	1,159	ND
	WHO-ISH, office	≥20%	35+	Y		19.8	20.2	8.1	24.0	10.4	4,715	2,198	SD
	WHO 2019, office	≥20%	40+	Y		21.7	22.6	8.5	25.2	10.8	6,935	1,555	SD
	Framingham, lab	≥20%	35+			19.8	17.5	8.1	26.3	10.8	5,519	1,957	SD
	WHO 2019, lab	≥10%	35+			19.8	18.7	8.1	27.0	10.9	5,927	1,833	SD
	WHO-ISH, office	≥20%	40+	Y		21.7	21.9	8.5	25.0	11.0	6,835	1,613	SD
	Framingham, office	≥20%	35+		Y	27.8	13.9	8.1	25.1	11.0	6,828	1,615	SD
	WHO-ISH, lab	≥10%	35+		Y	27.1	11.5	8.1	24.9	11.4	4,961	2,294	SD
	WHO-ISH, office	≥10%	35+	Y		19.8	21.8	8.1	24.0	11.7	5,560	2,103	SD
	WHO-ISH, lab	≥10%	40+		Y	29.3	13.0	8.5	25.8	12.1	7,177	1,689	SD
G	Globorisk, office	≥20%	40+		Y	30.9	17.0	8.5	27.2	12.3	10,182	1,206	ND
	WHO-ISH, office	≥10%	40+	Y		21.7	23.9	8.5	25.0	12.6	7,905	1,593	SD
	WHO 2019, office	≥20%	35+	Y	Y	26.8	20.6	8.1	24.1	13.0	6,314	2,052	SD
	WHO 2019, lab	≥10%	40+			21.7	22.4	8.5	28.8	13.2	8,735	1,509	SD
	Framingham, lab	≥20%	40+			21.7	20.9	8.5	27.8	13.2	8,253	1,596	SD
	WHO-ISH, office	≥20%	35+	Y	Y	26.7	20.2	8.1	24.0	13.3	6,344	2,091	SD
	WHO 2019, lab	≥20%	35+	Y		19.8	23.3	8.1	26.3	13.3	5,372	2,482	SD
	WHO-ISH, lab	≥20%	35+	Y		19.8	23.0	8.1	26.1	13.7	5,498	2,498	SD
	WHO 2019, office	≥20%	40+	Y	Y	29.1	22.6	8.5	25.2	13.7	8,848	1,554	SD
	Framingham, office	≥20%	40+		Y	30.3	17.7	8.5	26.9	13.7	9,990	1,375	SD
	WHO 2019, lab	≥20%	40+	Y		21.7	25.1	8.5	27.1	13.8	7,704	1,786	SD
	WHO-ISH, office	≥20%	40+	Y	Y	28.9	21.9	8.5	25.0	14.0	8,697	1,606	SD
	WHO-ISH, lab	≥20%	40+	Y		21.7	24.5	8.5	26.9	14.0	7,697	1,825	SD
	WHO 2019, office	≥10%	35+	Y		19.8	27.3	8.1	26.7	14.0	7,611	1,841	SD
	WHO-ISH, office	≥10%	35+	Y	Y	26.7	21.8	8.1	24.0	14.6	7,189	2,029	SD
	WHO 2019, lab	≥10%	35+		Y	28.2	18.7	8.1	28.0	14.8	8,209	1,808	SD
	Framingham, lab	≥20%	35+		Y	27.8	17.5	8.1	27.4	14.8	7,741	1,912	SD
	Globorisk, office	≥20%	35+	Y		19.8	28.3	8.1	26.6	15.2	8,140	1,867	SD
	Framingham, office	≥20%	35+	Y		19.8	26.4	8.1	26.4	15.3	7,309	2,100	SD
	WHO-ISH, office	≥10%	40+	Y	Y	28.9	23.9	8.5	25.0	15.5	9,767	1,590	SD
	WHO 2019, office	≥10%	40+	Y		21.7	31.1	8.5	28.5	15.8	10,565	1,494	ED
	WHO-ISH, lab	≥10%	35+	Y		19.8	25.6	8.1	26.1	16.2	6,575	2,470	SD
	WHO 2019, lab	≥20%	35+	Y	Y	26.7	23.3	8.1	26.3	16.3	7,025	2,316	SD
	WHO-ISH, lab	≥20%	35+	Y	Y	26.7	23.0	8.1	26.1	16.7	7,127	2,338	SD
	WHO 2019, lab	≥20%	40+	Y	Y	29.0	25.1	8.5	27.1	16.7	9,598	1,744	SD
	WHO-ISH, lab	≥10%	40+	Y		21.7	27.6	8.5	26.9	16.8	8,998	1,863	SD
	WHO-ISH, lab	≥20%	40+	Y	Y	28.9	24.5	8.5	26.9	17.0	9,558	1,781	SD
	WHO 2019, lab	≥10%	40+		Y	30.8	22.4	8.5	29.7	17.1	11,401	1,502	ED
	Framingham, lab	≥20%	40+		Y	30.4	20.9	8.5	28.9	17.1	10,840	1,580	SD
	WHO 2019, office	≥10%	35+	Y	Y	27.9	27.3	8.1	26.7	17.2	9,697	1,770	SD
	Globorisk, office	≥20%	40+	Y		21.7	32.3	8.5	28.4	17.2	11,209	1,537	ED
	Framingham, office	≥20%	40+	Y		21.7	30.1	8.5	28.1	17.5	10,322	1,697	SD
	Globorisk, lab	≥20%	35+			19.8	25.5	8.1	28.8	17.8	8,268	2,151	SD
	Framingham, office	≥20%	35+	Y	Y	27.8	26.4	8.1	26.4	18.5	9,382	1,967	SD
	Globorisk, office	≥20%	35+	Y	Y	28.3	28.3	8.1	26.6	18.5	10,387	1,777	SD
	WHO 2019, lab	≥10%	35+	Y		19.8	30.1	8.1	29.1	18.6	8,292	2,245	SD
	Framingham, lab	≥20%	35+	Y		19.8	29.3	8.1	28.7	18.6	8,102	2,300	SD
F	WHO 2019, office	≥10%	40+	Y	Y	30.5	31.1	8.5	28.5	19.0	13,010	1,464	ED
	WHO-ISH, lab	≥10%	35+	Y	Y	27.1	25.6	8.1	26.1	19.3	8,303	2,326	SD
	WHO-ISH, lab	≥10%	40+	Y	Y	29.3	27.6	8.5	26.9	19.9	10,972	1,811	SD
	WHO 2019, lab	≥10%	40+	Y		21.7	33.6	8.5	30.7	20.3	11,276	1,803	SD
	Framingham, lab	≥20%	40+	Y		21.7	32.6	8.5	30.1	20.4	11,046	1,846	SD
	Globorisk, lab	≥20%	40+			21.7	29.9	8.5	30.5	20.5	11,364	1,806	SD
	Globorisk, office	≥20%	40+	Y	Y	30.9	32.3	8.5	28.4	20.6	13,854	1,488	ED
	Framingham, office	≥20%	40+	Y	Y	30.3	30.1	8.5	28.1	20.7	12,752	1,626	SD
	Framingham, office	≥10%	35+			19.8	31.6	8.1	31.7	21.2	10,727	1,976	SD
	WHO 2019, lab	≥10%	35+	Y	Y	28.2	30.1	8.1	29.1	21.8	10,464	2,084	SD
	Framingham, lab	≥20%	35+	Y	Y	27.8	29.3	8.1	28.7	21.8	10,207	2,139	SD
	Globorisk, lab	≥20%	35+		Y	29.4	25.5	8.1	29.5	21.9	10,971	1,999	SD
	Globorisk, lab	≥20%	35+	Y		19.8	32.9	8.1	30.4	23.2	9,521	2,437	SD
	WHO 2019, lab	≥10%	40+	Y	Y	30.8	33.6	8.5	30.7	23.7	13,832	1,710	SD

Scenario	CVD risk tool	High CVD risk	Ages screened	Statins all diabetics	HTN medication at lower BP	% of screened people newly commenced on: Anti-hypertensive	Statin	Anti-diabetic	At least 1 medication	Incremental costs ($)	Incremental QALYs	ICER ($/QALY)	Dominance
	Framingham, lab	≥20%	40+	Y	Y	30.4	32.6	8.5	30.1	23.7	13,516	1,754	SD
	Globorisk, lab	≥20%	40+		Y	31.9	29.9	8.5	31.1	24.5	14,399	1,702	ED
	Globorisk, lab	≥20%	40+	Y		21.7	36.4	8.5	31.8	25.0	12,557	1,993	SD
	Globorisk, office	≥10%	35+			19.8	36.5	8.1	36.8	25.1	12,549	1,998	SD
	Framingham, office	≥10%	35+		Y	31.2	31.6	8.1	32.7	25.8	14,024	1,840	SD
	Framingham, lab	≥10%	35+			19.8	35.6	8.1	34.8	26.5	11,557	2,291	SD
	Globorisk, lab	≥20%	35+	Y	Y	29.4	32.9	8.1	30.4	26.8	12,159	2,201	SD
	Framingham, office	≥10%	35+	Y		19.8	39.3	8.1	33.8	26.9	12,044	2,231	SD
	Framingham, office	≥10%	40+			21.7	40.0	8.5	35.7	27.5	14,968	1,835	ED
	Globorisk, lab	≥20%	40+	Y	Y	31.9	36.4	8.5	31.8	28.6	15,536	1,840	ED
	Globorisk, office	≥10%	40+			21.7	43.7	8.5	40.4	29.7	16,445	1,806	ED
	Globorisk, office	≥10%	35+		Y	33.7	36.5	8.1	37.6	30.7	16,616	1,850	ED
	Framingham, office	≥10%	35+	Y	Y	31.2	39.3	8.1	33.8	30.9	15,272	2,021	SD
	Framingham, lab	≥10%	35+		Y	32.0	35.6	8.1	35.7	31.5	15,063	2,090	SD
	Globorisk, office	≥10%	35+	Y		19.8	45.9	8.1	38.6	31.7	14,178	2,236	SD
	Framingham, lab	≥10%	35+	Y		19.8	42.6	8.1	36.8	31.8	12,753	2,492	SD
	Framingham, office	≥10%	40+	Y		21.7	46.3	8.5	37.5	32.0	16,160	1,977	SD
	Framingham, lab	≥10%	40+			21.7	43.1	8.5	38.0	32.0	15,604	2,052	SD
	Framingham, office	≥10%	40+		Y	34.7	40.0	8.5	36.5	32.2	18,920	1,703	ED
H	Globorisk, office	≥10%	40+		Y	36.7	43.7	8.5	41.1	35.3	21,002	1,681	ND
	Globorisk, office	≥10%	40+	Y		21.7	52.2	8.5	41.9	35.4	18,036	1,963	SD
	Framingham, lab	≥10%	35+	Y	Y	32.0	42.6	8.1	36.8	36.1	16,193	2,232	SD
	Framingham, lab	≥10%	40+	Y		21.7	48.7	8.5	39.7	36.1	16,649	2,166	SD
	Framingham, office	≥10%	40+	Y	Y	34.7	46.3	8.5	37.5	36.3	20,055	1,809	SD
	Globorisk, office	≥10%	35+	Y	Y	33.7	45.9	8.1	38.6	36.7	18,163	2,022	SD
	Framingham, lab	≥10%	40+		Y	35.1	43.1	8.5	38.7	37.0	19,634	1,883	SD
	Globorisk, lab	≥10%	35+			19.8	48.1	8.1	42.1	39.7	14,792	2,682	SD
I	Globorisk, office	≥10%	40+	Y	Y	36.7	52.2	8.5	41.9	40.5	22,518	1,799	ND
	Framingham, lab	≥10%	40+	Y	Y	35.1	48.7	8.5	39.7	40.6	20,628	1,968	SD
	Globorisk, lab	≥10%	35+	Y		19.8	51.3	8.1	43.1	42.2	15,236	2,771	SD
	Globorisk, lab	≥10%	40+			21.7	54.1	8.5	44.5	42.9	18,496	2,318	SD
	Globorisk, lab	≥10%	40+	Y		21.7	56.2	8.5	45.2	44.5	18,808	2,364	SD
	Globorisk, lab	≥10%	35+		Y	34.9	48.1	8.1	42.6	45.4	19,017	2,385	SD
	Globorisk, lab	≥10%	35+	Y	Y	34.9	51.3	8.1	43.1	47.6	19,436	2,449	SD
	Globorisk, lab	≥10%	40+		Y	37.9	54.1	8.5	44.7	48.4	23,216	2,086	ED
J	Globorisk, lab	≥10%	40+	Y	Y	37.9	56.2	8.5	45.2	49.9	23,514	2,121	ND

Notes: Scenario labels as used in Figure 3 and Additional file 1: Figures S3-S8. HTN = hypertension, Y = Yes, ND = Not dominated, SD = Dominated (strong dominance), ED = Dominated (extended dominance), CS = Cost saving.

Table S4 Incremental costs, QALYs and ICERs of all models (sorted by scenario)

Scenario	CVD risk tool	High CVD risk	Ages screened	Statins all diabetics	HTN medication at lower BP	% of screened people newly commenced on: Anti-hypertensive	Statin	Anti-diabetic	At least 1 medication	Incremental costs ($)	Incremental QALYs	ICER ($/QALY)	Dominance
C	WHO-ISH, office	≥30%	40-65			21.2	0.9	8.8	22.3	1.5	-153	-9,538	SD
Base	WHO-ISH, office	≥20%	35+			19.8	2.4	8.1	21.2	0.0	0		Base
	WHO-ISH, office	≥20%	35+		Y	26.7	2.4	8.1	22.6	3.8	1,818	2,072	SD
	WHO-ISH, office	≥20%	35+	Y		19.8	20.2	8.1	24.0	10.4	4,715	2,198	SD
	WHO-ISH, office	≥20%	35+	Y	Y	26.7	20.2	8.1	24.0	13.3	6,344	2,091	SD
	WHO-ISH, office	≥20%	40+			21.7	2.8	8.5	22.3	0.7	1,362	481	ND
	WHO-ISH, office	≥20%	40+		Y	28.9	2.8	8.5	23.7	4.3	3,431	1,268	SD
	WHO-ISH, office	≥20%	40+	Y		21.7	21.9	8.5	25.0	11.0	6,835	1,613	SD
	WHO-ISH, office	≥20%	40+	Y	Y	28.9	21.9	8.5	25.0	14.0	8,697	1,606	SD
	WHO-ISH, office	≥10%	35+			19.8	6.1	8.1	21.3	2.0	1,593	1,274	ED
	WHO-ISH, office	≥10%	35+		Y	26.7	6.1	8.1	22.6	5.8	3,401	1,698	SD
	WHO-ISH, office	≥10%	35+	Y		19.8	21.8	8.1	24.0	11.7	5,560	2,103	SD
	WHO-ISH, office	≥10%	35+	Y	Y	26.7	21.8	8.1	24.0	14.6	7,189	2,029	SD
	WHO-ISH, office	≥10%	40+			21.7	7.3	8.5	22.4	3.0	3,344	910	ED
	WHO-ISH, office	≥10%	40+		Y	28.9	7.3	8.5	23.7	6.7	5,401	1,242	ND
	WHO-ISH, office	≥10%	40+	Y		21.7	23.9	8.5	25.0	12.6	7,905	1,593	SD
	WHO-ISH, office	≥10%	40+	Y	Y	28.9	23.9	8.5	25.0	15.5	9,767	1,590	SD
	WHO-ISH, lab	≥20%	35+			19.8	6.4	8.1	23.5	3.9	1,076	3,649	SD
	WHO-ISH, lab	≥20%	35+		Y	26.7	6.4	8.1	24.8	7.8	2,883	2,694	SD
	WHO-ISH, lab	≥20%	35+	Y		19.8	23.0	8.1	26.1	13.7	5,498	2,498	SD
	WHO-ISH, lab	≥20%	35+	Y	Y	26.7	23.0	8.1	26.1	16.7	7,127	2,338	SD
	WHO-ISH, lab	≥20%	40+			21.7	6.7	8.5	24.3	4.3	2,572	1,682	SD
	WHO-ISH, lab	≥20%	40+		Y	28.9	6.7	8.5	25.6	8.1	4,628	1,746	SD
	WHO-ISH, lab	≥20%	40+	Y		21.7	24.5	8.5	26.9	14.0	7,697	1,825	SD
	WHO-ISH, lab	≥20%	40+	Y	Y	28.9	24.5	8.5	26.9	17.0	9,558	1,781	SD
	WHO-ISH, lab	≥10%	35+			19.8	11.5	8.1	23.7	7.4	3,068	2,422	SD
	WHO-ISH, lab	≥10%	35+		Y	27.1	11.5	8.1	24.9	11.4	4,961	2,294	SD
	WHO-ISH, lab	≥10%	35+	Y		19.8	25.6	8.1	26.1	16.2	6,575	2,470	SD
	WHO-ISH, lab	≥10%	35+	Y	Y	27.1	25.6	8.1	26.1	19.3	8,303	2,326	SD
	WHO-ISH, lab	≥10%	40+			21.7	13.0	8.5	24.6	8.3	5,025	1,647	SD
	WHO-ISH, lab	≥10%	40+		Y	29.3	13.0	8.5	25.8	12.1	7,177	1,689	SD
	WHO-ISH, lab	≥10%	40+	Y		21.7	27.6	8.5	26.9	16.8	8,998	1,863	SD
	WHO-ISH, lab	≥10%	40+	Y	Y	29.3	27.6	8.5	26.9	19.9	10,972	1,811	SD
A	WHO 2019, office	≥20%	35+			19.8	2.1	8.1	21.4	-0.6	-426		CS
	WHO 2019, office	≥20%	35+		Y	26.8	2.1	8.1	22.7	3.2	1,429	2,249	SD
	WHO 2019, office	≥20%	35+	Y		19.8	20.6	8.1	24.1	10.0	4,646	2,161	SD
	WHO 2019, office	≥20%	35+	Y	Y	26.8	20.6	8.1	24.1	13.0	6,314	2,052	SD
B	WHO 2019, office	≥20%	40+			21.7	2.7	8.5	22.5	0.1	1,007	113	ND
	WHO 2019, office	≥20%	40+		Y	29.1	2.7	8.5	23.9	3.8	3,123	1,225	SD
	WHO 2019, office	≥20%	40+	Y		21.7	22.6	8.5	25.2	10.8	6,935	1,555	SD
	WHO 2019, office	≥20%	40+	Y	Y	29.1	22.6	8.5	25.2	13.7	8,848	1,554	SD
	WHO 2019, office	≥10%	35+			19.8	11.4	8.1	24.2	4.2	3,708	1,146	ED
	WHO 2019, office	≥10%	35+		Y	27.9	11.4	8.1	25.4	8.2	5,957	1,380	SD
	WHO 2019, office	≥10%	35+	Y		19.8	27.3	8.1	26.7	14.0	7,611	1,841	SD
	WHO 2019, office	≥10%	35+	Y	Y	27.9	27.3	8.1	26.7	17.2	9,697	1,770	SD
D	WHO 2019, office	≥10%	40+			21.7	14.5	8.5	26.1	6.2	6,129	1,009	ND
E	WHO 2019, office	≥10%	40+		Y	30.5	14.5	8.5	27.2	10.1	8,747	1,159	ND
	WHO 2019, office	≥10%	40+	Y		21.7	31.1	8.5	28.5	15.8	10,565	1,494	ED
F	WHO 2019, office	≥10%	40+	Y	Y	30.5	31.1	8.5	28.5	19.0	13,010	1,464	ED
	WHO 2019, lab	≥20%	35+			19.8	7.3	8.1	23.7	3.7	1,225	2,985	SD
	WHO 2019, lab	≥20%	35+		Y	26.7	7.3	8.1	25.0	7.5	3,044	2,457	SD
	WHO 2019, lab	≥20%	35+	Y		19.8	23.3	8.1	26.3	13.3	5,372	2,482	SD
	WHO 2019, lab	≥20%	35+	Y	Y	26.7	23.3	8.1	26.3	16.3	7,025	2,316	SD
	WHO 2019, lab	≥20%	40+			21.7	8.2	8.5	24.6	4.2	2,939	1,430	SD
	WHO 2019, lab	≥20%	40+		Y	29.0	8.2	8.5	25.8	7.9	5,011	1,583	SD
	WHO 2019, lab	≥20%	40+	Y		21.7	25.1	8.5	27.1	13.8	7,704	1,786	SD
	WHO 2019, lab	≥20%	40+	Y	Y	29.0	25.1	8.5	27.1	16.7	9,598	1,744	SD
	WHO 2019, lab	≥10%	35+			19.8	18.7	8.1	27.0	10.9	5,927	1,833	SD
	WHO 2019, lab	≥10%	35+		Y	28.2	18.7	8.1	28.0	14.8	8,209	1,808	SD
	WHO 2019, lab	≥10%	35+	Y		19.8	30.1	8.1	29.1	18.6	8,292	2,245	SD
	WHO 2019, lab	≥10%	35+	Y	Y	28.2	30.1	8.1	29.1	21.8	10,464	2,084	SD
	WHO 2019, lab	≥10%	40+			21.7	22.4	8.5	28.8	13.2	8,735	1,509	SD
	WHO 2019, lab	≥10%	40+		Y	30.8	22.4	8.5	29.7	17.1	11,401	1,502	ED
	WHO 2019, lab	≥10%	40+	Y		21.7	33.6	8.5	30.7	20.3	11,276	1,803	SD
	WHO 2019, lab	≥10%	40+	Y	Y	30.8	33.6	8.5	30.7	23.7	13,832	1,710	SD
	Framingham, office	≥20%	35+			19.8	13.9	8.1	23.9	7.1	4,628	1,544	SD
	Framingham, office	≥20%	35+		Y	27.8	13.9	8.1	25.1	11.0	6,828	1,615	SD
	Framingham, office	≥20%	35+	Y		19.8	26.4	8.1	26.4	15.3	7,309	2,100	SD
	Framingham, office	≥20%	35+	Y	Y	27.8	26.4	8.1	26.4	18.5	9,382	1,967	SD
	Framingham, office	≥20%	40+			21.7	17.7	8.5	25.8	9.9	7,433	1,332	ED
	Framingham, office	≥20%	40+		Y	30.3	17.7	8.5	26.9	13.7	9,990	1,375	SD
	Framingham, office	≥20%	40+	Y		21.7	30.1	8.5	28.1	17.5	10,322	1,697	SD
	Framingham, office	≥20%	40+	Y	Y	30.3	30.1	8.5	28.1	20.7	12,752	1,626	SD
	Framingham, office	≥10%	35+			19.8	31.6	8.1	31.7	21.2	10,727	1,976	SD
	Framingham, office	≥10%	35+		Y	31.2	31.6	8.1	32.7	25.8	14,024	1,840	SD
	Framingham, office	≥10%	35+	Y		19.8	39.3	8.1	33.8	26.9	12,044	2,231	SD
	Framingham, office	≥10%	35+	Y	Y	31.2	39.3	8.1	33.8	30.9	15,272	2,021	SD
	Framingham, office	≥10%	40+			21.7	40.0	8.5	35.7	27.5	14,968	1,835	ED
	Framingham, office	≥10%	40+		Y	34.7	40.0	8.5	36.5	32.2	18,920	1,703	ED
	Framingham, office	≥10%	40+	Y		21.7	46.3	8.5	37.5	32.0	16,160	1,977	SD
	Framingham, office	≥10%	40+	Y	Y	34.7	46.3	8.5	37.5	36.3	20,055	1,809	SD
	Framingham, lab	≥20%	35+			19.8	17.5	8.1	26.3	10.8	5,519	1,957	SD
	Framingham, lab	≥20%	35+		Y	27.8	17.5	8.1	27.4	14.8	7,741	1,912	SD
	Framingham, lab	≥20%	35+	Y		19.8	29.3	8.1	28.7	18.6	8,102	2,300	SD
	Framingham, lab	≥20%	35+	Y	Y	27.8	29.3	8.1	28.7	21.8	10,207	2,139	SD
	Framingham, lab	≥20%	40+			21.7	20.9	8.5	27.8	13.2	8,253	1,596	SD
	Framingham, lab	≥20%	40+		Y	30.4	20.9	8.5	28.9	17.1	10,840	1,580	SD
	Framingham, lab	≥20%	40+	Y		21.7	32.6	8.5	30.1	20.4	11,046	1,846	SD
	Framingham, lab	≥20%	40+	Y	Y	30.4	32.6	8.5	30.1	23.7	13,516	1,754	SD
	Framingham, lab	≥10%	35+			19.8	35.6	8.1	34.8	26.5	11,557	2,291	SD
	Framingham, lab	≥10%	35+		Y	32.0	35.6	8.1	35.7	31.5	15,063	2,090	SD
	Framingham, lab	≥10%	35+	Y		19.8	42.6	8.1	36.8	31.8	12,753	2,492	SD
	Framingham, lab	≥10%	35+	Y	Y	32.0	42.6	8.1	36.8	36.1	16,193	2,232	SD

Scenario	CVD risk tool	High CVD risk	Ages screened	Statins all diabetics	HTN medication at lower BP	% of screened people newly commenced on:				Incremental costs ($)	Incremental QALYs	ICER ($/QALY)	Dominance
						Anti-hypertensive	Statin	Anti-diabetic	At least 1 medication				
	Framingham, lab	≥10%	40+			21.7	43.1	8.5	38.0	32.0	15,604	2,052	SD
	Framingham, lab	≥10%	40+		Y	35.1	43.1	8.5	38.7	37.0	19,634	1,883	SD
	Framingham, lab	≥10%	40+	Y		21.7	48.7	8.5	39.7	36.1	16,649	2,166	SD
	Framingham, lab	≥10%	40+	Y	Y	35.1	48.7	8.5	39.7	40.6	20,628	1,968	SD
	Globorisk, office	≥20%	35+			19.8	13.5	8.1	24.2	5.9	4,722	1,250	ED
	Globorisk, office	≥20%	35+		Y	28.3	13.5	8.1	25.3	10.0	7,113	1,400	SD
	Globorisk, office	≥20%	35+	Y		19.8	28.3	8.1	26.6	15.2	8,140	1,867	SD
	Globorisk, office	≥20%	35+	Y	Y	28.3	28.3	8.1	26.6	18.5	10,387	1,777	SD
	Globorisk, office	≥20%	40+			21.7	17.0	8.5	26.1	8.2	7,390	1,112	ED
G	Globorisk, office	≥20%	40+		Y	30.9	17.0	8.5	27.2	12.3	10,182	1,206	ND
	Globorisk, office	≥20%	40+	Y		21.7	32.3	8.5	28.4	17.2	11,209	1,537	ED
	Globorisk, office	≥20%	40+	Y	Y	30.9	32.3	8.5	28.4	20.6	13,854	1,488	ED
	Globorisk, office	≥10%	35+			19.8	36.5	8.1	36.8	25.1	12,549	1,998	SD
	Globorisk, office	≥10%	35+		Y	33.7	36.5	8.1	37.6	30.7	16,616	1,850	ED
	Globorisk, office	≥10%	35+	Y		19.8	45.9	8.1	38.6	31.7	14,178	2,236	SD
	Globorisk, office	≥10%	35+	Y	Y	33.7	45.9	8.1	38.6	36.7	18,163	2,022	SD
	Globorisk, office	≥10%	40+			21.7	43.7	8.5	40.4	29.7	16,445	1,806	ED
H	Globorisk, office	≥10%	40+		Y	36.7	43.7	8.5	41.1	35.3	21,002	1,681	ND
	Globorisk, office	≥10%	40+	Y		21.7	52.2	8.5	41.9	35.4	18,036	1,963	SD
I	Globorisk, office	≥10%	40+	Y	Y	36.7	52.2	8.5	41.9	40.5	22,518	1,799	ND
	Globorisk, lab	≥20%	35+			19.8	25.5	8.1	28.8	17.8	8,268	2,151	SD
	Globorisk, lab	≥20%	35+		Y	29.4	25.5	8.1	29.5	21.9	10,971	1,999	SD
	Globorisk, lab	≥20%	35+	Y		19.8	32.9	8.1	30.4	23.2	9,521	2,437	SD
	Globorisk, lab	≥20%	35+	Y	Y	29.4	32.9	8.1	30.4	26.8	12,159	2,201	SD
	Globorisk, lab	≥20%	40+			21.7	29.9	8.5	30.5	20.5	11,364	1,806	SD
	Globorisk, lab	≥20%	40+		Y	31.9	29.9	8.5	31.1	24.5	14,399	1,702	ED
	Globorisk, lab	≥20%	40+	Y		21.7	36.4	8.5	31.8	25.0	12,557	1,993	SD
	Globorisk, lab	≥20%	40+	Y	Y	31.9	36.4	8.5	31.8	28.6	15,536	1,840	ED
	Globorisk, lab	≥10%	35+			19.8	48.1	8.1	42.1	39.7	14,792	2,682	SD
	Globorisk, lab	≥10%	35+		Y	34.9	48.1	8.1	42.6	45.4	19,017	2,385	SD
	Globorisk, lab	≥10%	35+	Y		19.8	51.3	8.1	43.1	42.2	15,236	2,771	SD
	Globorisk, lab	≥10%	35+	Y	Y	34.9	51.3	8.1	43.1	47.6	19,436	2,449	SD
	Globorisk, lab	≥10%	40+			21.7	54.1	8.5	44.5	42.9	18,496	2,318	SD
	Globorisk, lab	≥10%	40+		Y	37.9	54.1	8.5	44.7	48.4	23,216	2,086	ED
	Globorisk, lab	≥10%	40+	Y		21.7	56.2	8.5	45.2	44.5	18,808	2,364	SD
J	Globorisk, lab	≥10%	40+	Y	Y	37.9	56.2	8.5	45.2	49.9	23,514	2,121	ND

Notes: This table is identical to Additional file 1: Table S3, however, rows are sorted by scenario, that is, by the columns CVD risk tool, High CVD risk, Ages screened, Statins all diabetics, and HTN medication at lower BP. Model labels as used in Figure 3 and Additional file 1: Figures S3-S8. HTN = hypertension, Y = Yes, ND = Not dominated, SD = Dominated (strong dominance), ED = Dominated (extended dominance), CS = Cost saving.

Figure S1 Comparison of percentage of people who will develop cardiovascular disease between 2019-2028 by age and prediction tool

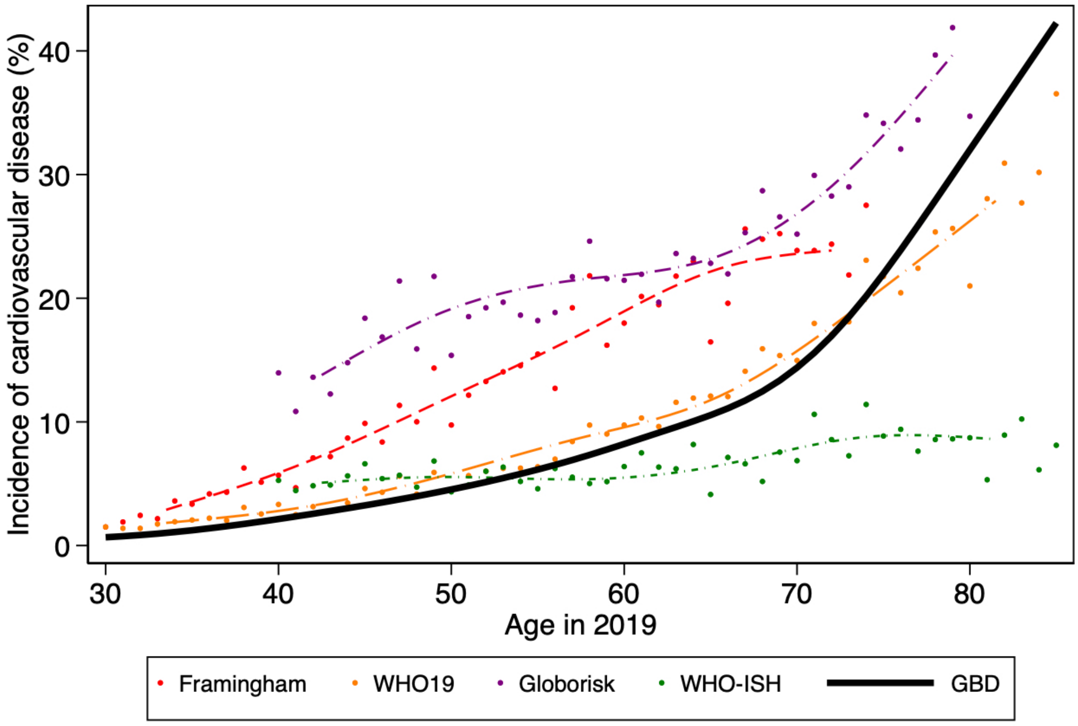

Notes: Figure S1 plots the predicted incidence of CVD over 10 years by age in 2019 using the WHO-ISH, WHO-2019, Globorisk and Framingham tools and SLHAS data, and compares it to the incidence of CVD obtained from extrapolating GBD incidence data.

GBD data for the incidence of ischaemic heart disease (GBD category 493) and stroke (GBD category 494) between 2010 and 2019 by 5-year age groups and gender were used to extrapolate the incident cases that were expected to happen from 2019 – 2029 by age group and gender. Cubic splines were fitted for each gender to smooth the 5-year age groups. The incident cases of ischaemic heart disease and stroke were summed to produce the incidence of cardiovascular disease between 2019 – 2028.

The predicted incidence of cardiovascular disease in the following 10 years (i.e. approximately 2019-2028) was calculated for each of the WHO-ISH, WHO-2019, Globorisk and Framingham tools using weighted data from SLHAS participants.

The predicted incidence using WHO-2019 most closely follows the predicted incidence using GBD data. There is a limitation to this crude analysis: the WHO-2019 tool uses GBD regional incidence data for IHD and stroke for calibration, so it is not unexpected that the prediction using the WHO-2019 tool fits the GBD data best. However, there is a lack of an alternative data source to estimate incident CVD events.

Figure S2 Mortality rates by age and gender, for coronary heart disease and stroke

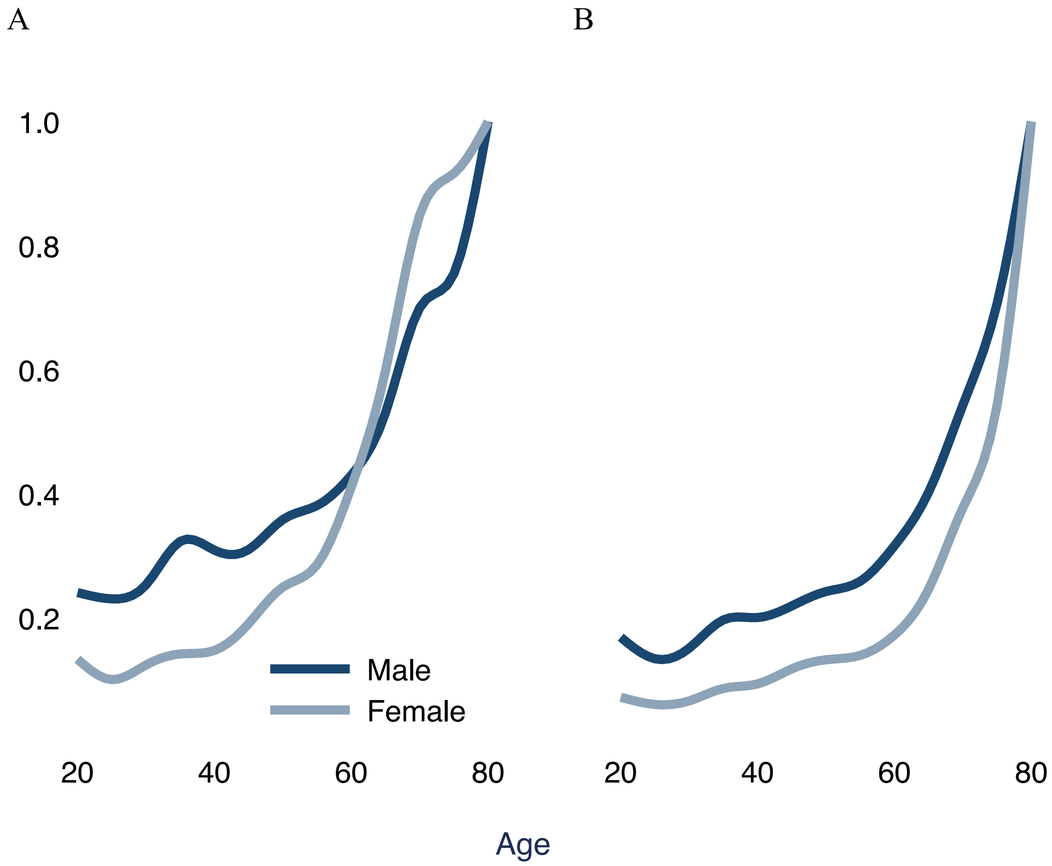

Notes: A. Coronary heart disease B. Stroke

Figure S3 Comparison of ICERs of the current protocol, and proposed scenarios using the WHO-2019 lab tool versus WHO-2019 office tool

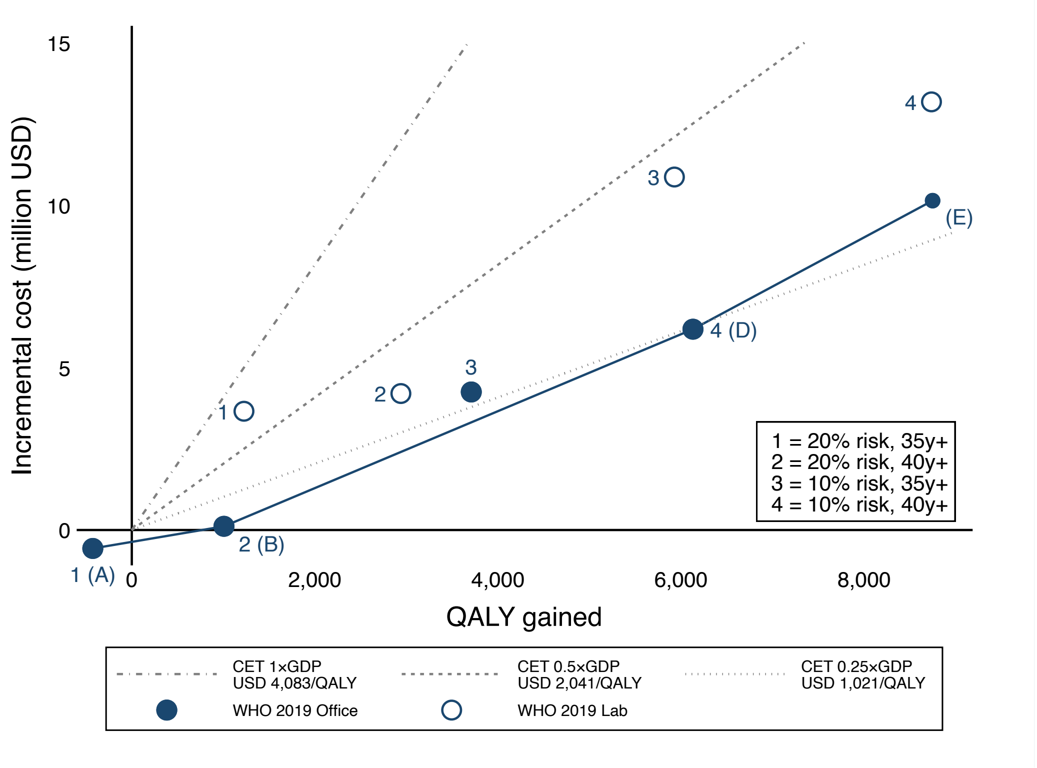

Notes: Scenarios A, B, D and E on the cost-effectiveness frontier shown in Table 2 and Figure 3 are shown.

Figure S4 Cost-effectiveness frontier - LBP and SD for WHO 2019 office risk tool

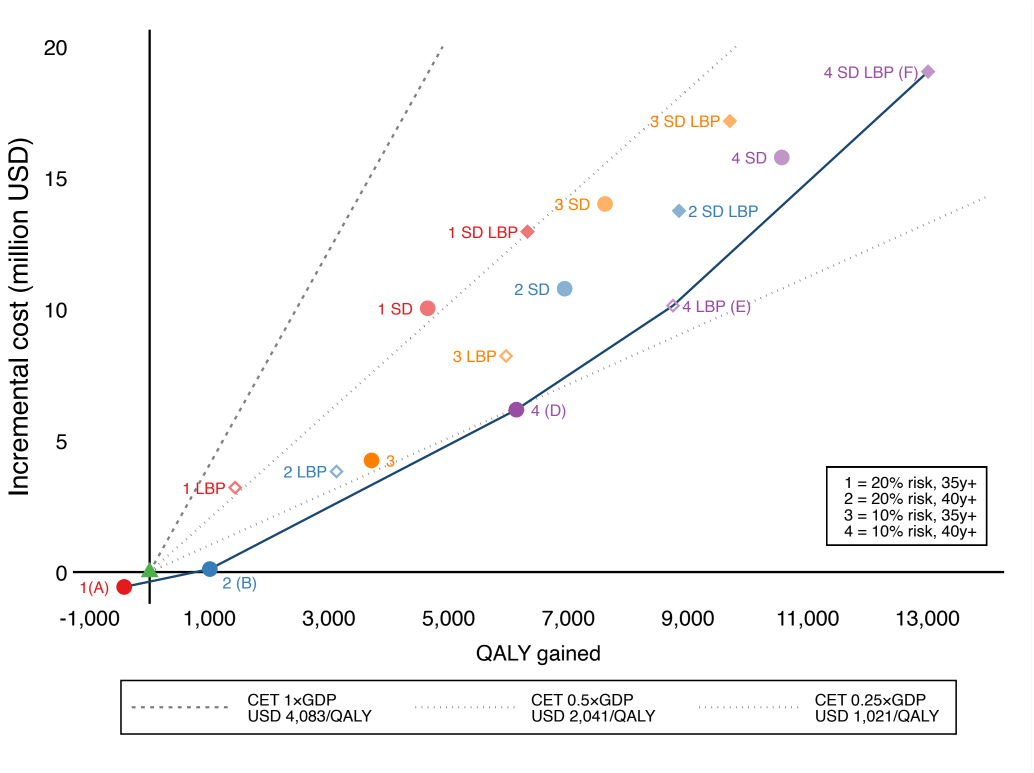

Notes: All scenarios used the WHO-2019 office tool. LBP = lowered blood pressure threshold, SD = statins for diabetics. Scenarios A, B, D, E, F on the cost-effectiveness frontier shown in Table 2 and Figure 3 are shown.

Figure S5 Comparison of ICERs of the old program, and proposed programs using any risk tool, and modifying risk thresholds and age-groups screened

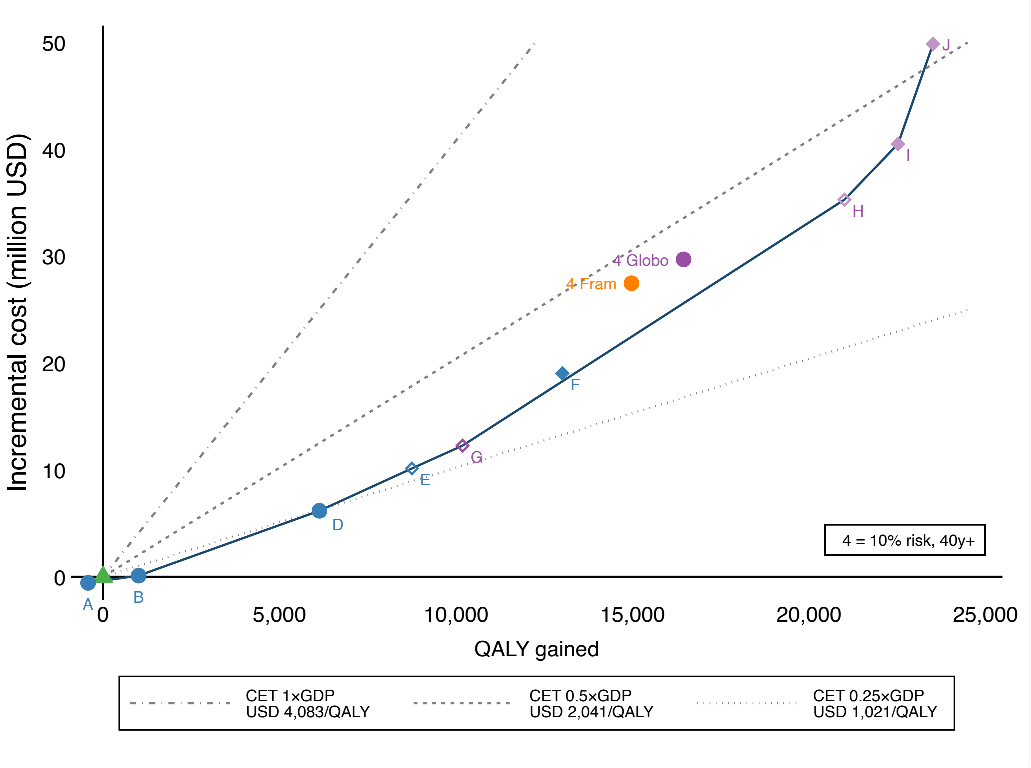

Notes: Globo = Globorisk, Fram = Framingham. Triangles denote scenarios that used the WHO-ISH office tool. Hollow diamonds are tools that included LBP. Filled diamonds are tools that included SD and LBP. All risk tools were office based except for scenario L. Scenarios A, B, D, E, and F, which are shown in Table 2 and Figure 3 are on the cost-effectiveness frontier when considering scenarios which only use WHO-ISH and WHO-2019. When scenarios that used Globorisk and Framingham tools were included, only Scenario F was no longer on the cost-effectiveness frontier. Additionally, Globorisk scenarios G, H, I, and J were on the new cost-effectiveness frontier.
Scenario G: Globorisk office, 20%, 40+, LBP
Scenario H: Globorisk office, 10%, 40+, LBP
Scenario I: Globorisk office, 10%, 40+, SD, LBP
Scenario J: Globorisk lab, 10%, 40+, SD, LBP

Scenarios H, I, J using Globorisk with a 10% threshold lie on the cost-effectiveness frontier and have the highest QALYs gained out of all 129 scenarios modelled. Compared to the base case, Scenarios H and I cost below 0.5×GDP per capita/QALY, and Scenario J costs below 1×GDP per capita/QALY. However, moving from Scenario H to I and I to J costs 0.8 and 2.3×GDP per capita/QALY, respectively.

Figure S6 Probabilistic sensitivity analysis of scenarios on the cost-effectiveness frontier

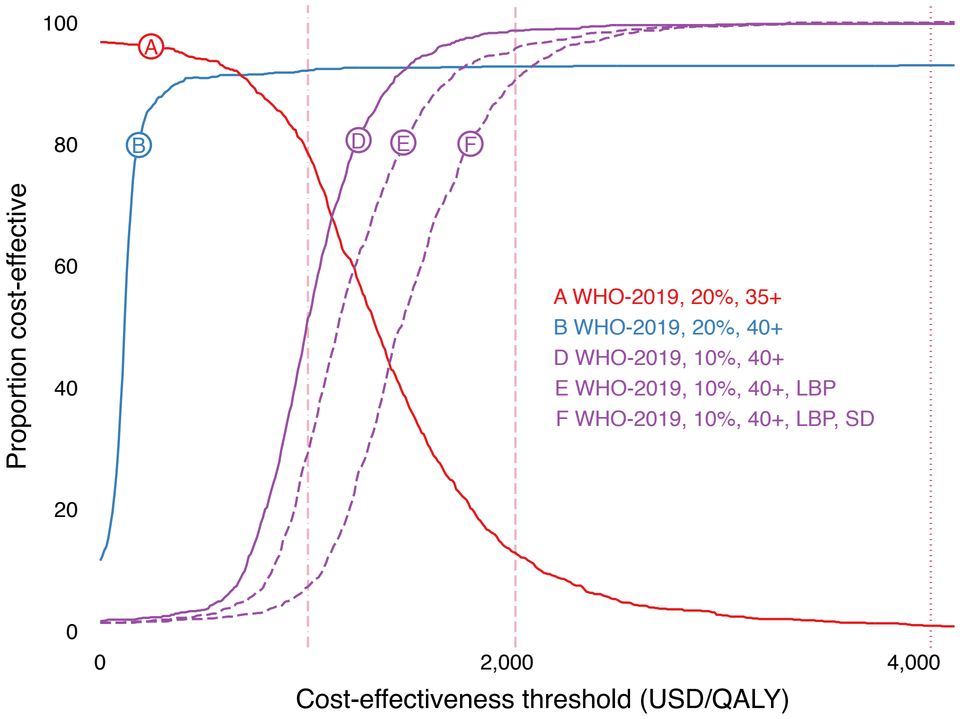

Notes: The probabilistic sensitivity traces are shown for scenarios A, B, D, E, and F, which were on the cost-effectiveness frontier (Figure 3). Dotted vertical lines show CET thresholds for 1 QALY at 0.25 (USD 1,021), 0.5 (USD 2,041) and 1 (USD 4,083)×GDP per capita.

Figure S7 Impact on cost, impact and cost-effectiveness frontier, when including pill disutility

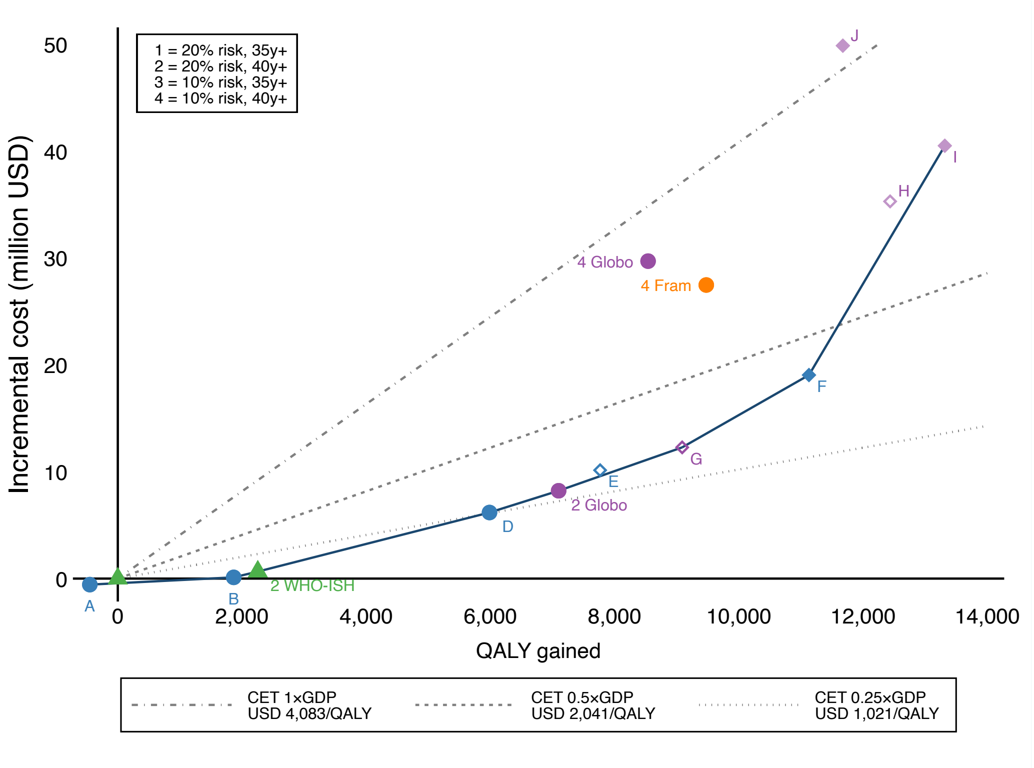

Notes: Globo = Globorisk, Fram = Framingham. Triangles denote scenarios that used the WHO-ISH office tool. Hollow diamonds are scenarios that included LBP; filled diamonds are scenarios that used SD and LBP.

Scenarios A, B, D, G and I, which are on the cost-effectiveness frontier when considering all screening tools (Additional file 1: Figure S5) remained on the frontier. However, scenarios E and H have moved slightly away from the frontier, and scenario J has moved well away from the frontier. Three programs have moved onto the frontier: F, and two additional programs labelled as 2 WHO-ISH (WHO-ISH Office, 20%, 40+) and 2 Globo (Globorisk Office, 20%, 40+).

Figure S8 Incremental costs and QALYs by age category for Scenario G (WHO-2019 office, 10% threshold, 40+, SD and LBP) model compared to the base-case

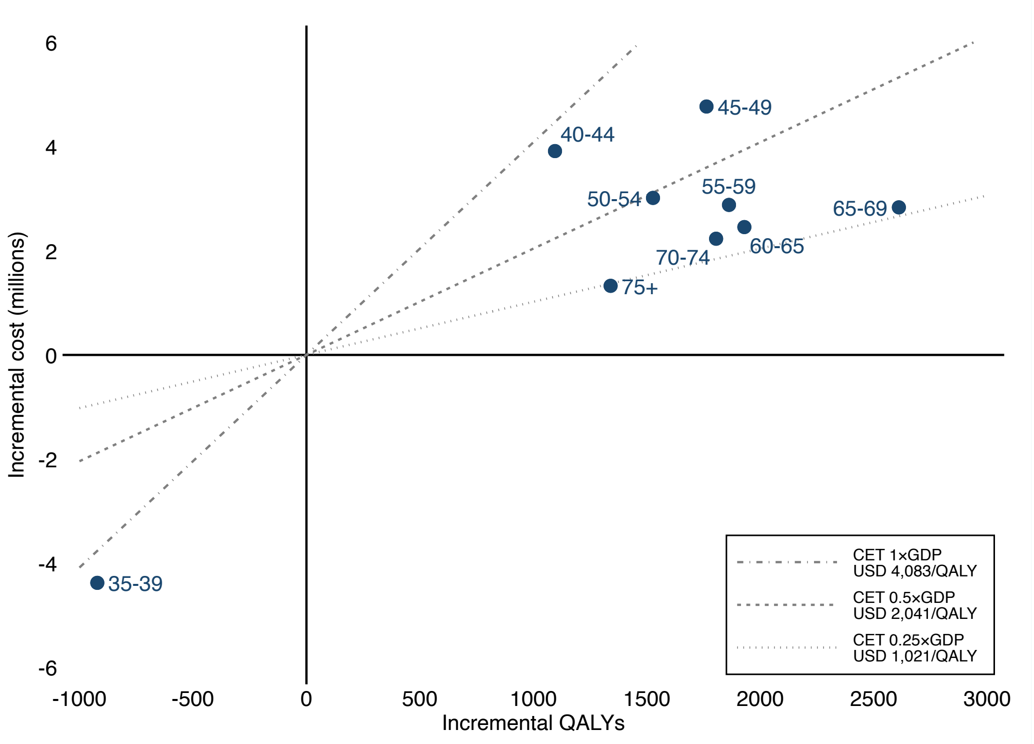

Notes: Age group 35-39 had negative QALYs and incremental cost as scenario F did not screen the 35-39 age group, and the base case did.

Text S1

Costs of medicines were calculated from data obtained from the Medical Supplies Division of the Ministry of Health [1]. Laboratory costs were based on prices for reagents, consumables, and labour cost in the public sector in 2019. The cost of a consultation was calculating by dividing total public expenditure on outpatient care by the number of outpatient visits in 2019 [2, 3]. We obtained the cost per hospital admission for each of CHD and stroke from a 2005 Sri Lanka public hospital survey of condition-specific costs and admissions [2, 4]. We inflated these costs to 2019 values using the 2019:2005 ratio of total public inpatient expenditures.

Costs of usual care

We also allocated a cost of usual medical care to all individuals based on an estimated average cost of inpatient and outpatient care by 10-year age groups using data from multiple sources [2-4]. Usual costs for people with incident CHD or stroke in the first 10 years were increased by factors based on analysis of inpatient and outpatient contacts in people with CHD and stroke compared to those without.

Outpatient care

The total current expenditure on public outpatient care was calculated by applying the share of outpatient expenditure that is public (36%) to the total outpatient expenditure (Rs. 89,242 million) in 2019 [2, 3]. Since some of this expenditure would be on people less than 18 years old, we adjusted the expenditure by the ratio of non-paediatric clinic visits to all clinic visits (0.98).

We used SLHAS Wave 1 data to estimate the weighted distribution of outpatient visits to the public sector by 10-year age groups (Additional file 1: Table S5). The adult public expenditure on outpatient care was distributed amongst each age group based on

each age group's proportion of outpatient visits. The total cost of each age group was divided by the estimated population of that age group to estimate an average annual cost for outpatient care per person of that age group [5].

Inpatient care

The total current expenditure on public inpatient care was calculated by applying the share of inpatient expenditure that is public (74%) to the total inpatient expenditure (Rs. 207,258 million) in 2019 [2, 3]. To exclude expenditures on people less than 18 years old, we used the percentage of bed-days used by people aged 18 years and over from the Public Hospital Inpatient Discharge Survey (PHIDS) (77%) [4]

Similar to the technique used for outpatient care, we used SLHAS Wave 1 data to estimate the weighted distribution of inpatient visits to the public sector by 10-year age group, allocated total costs for each age group, and used the estimated population for each age group to estimate an average annual cost for inpatient care [5] (Additional file 1: Table S5).

Table S5 Distribution of inpatient and outpatient encounters, costs, and cost per capita

Age group	Distribution of visits	Total costs (million LKR)	Cost per capita (LKR)	Cost per capita (USD)
**Outpatient**				
18-24	12.49	3,920	1,748	9.6
25-34	10.04	3,151	1,064	5.9
35-44	17.47	5,483	1,786	9.8
45-54	16.13	5,063	1,938	10.7
55-64	20.67	6,488	2,853	15.7
65-74	17.33	5,440	3,544	19.5
≥ 75	5.88	1,846	2,537	14.0
**Inpatient**				
18-24	12.03	14,295	6,373	35.1
25-34	15.08	17,919	6,051	33.3
35-44	16.4	19,487	6,348	34.9
45-54	18.79	22,327	8,547	47.1
55-64	16.52	19,630	8,631	47.5
65-74	13.83	16,433	10,707	58.9
≥75	7.35	8,734	12,007	66.1

Adjusting costs for people with CHD and stroke

The SLHAS Wave 1 data collected information on number of inpatient and outpatient visits based on patient recall [6]. Data were also collected on whether participants had CHD and stroke based on self-report and medical records. Annualised inpatient and outpatient numbers were calculated for all participants.

Negative binomial regressions were run for inpatient and outpatient encounters respectively, to determine the impact of having CHD and stroke on number of encounters, after controlling for age, gender and socioeconomic quintile. The coefficients were exponentiated to produce factors to increase annual usual inpatient and outpatient costs for individuals with CHD or stroke. The exponentiated values are also presented in Table S6.

Table S6 Coefficients and 95% confidence intervals of negative binomial regression to assess the impact of CHD and stroke on inpatient and outpatient encounter numbers

	Log values		Exponentiated values	
	**Coefficient**	**95% CI**	**Coefficient**	**95% CI**
**Inpatient**				
CHD	1.05	(0.58 - 1.51)	2.85	(1.79 - 4.54)
Stroke	0.09	(-0.64 - 0.82)	1.09	(0.53 - 2.26)
**Outpatient**				
CHD	0.67	(0.37 - 0.96)	1.95	(1.45 - 2.61)
Stroke	0.68	(-0.19 - 1.54)	1.97	(0.83 - 4.69)

References

1. Institute for Health Policy. Analysis of database of pharmaceuticals issued by the Medical Supplies Division (MSD), Ministry of Health, Sri Lanka, between 2005-2019. Colombo, 2021.
2. Amarasinghe SN, Dalpatadu KCS, Rannan-Eliya RP. Sri Lanka Health Accounts: National Health Expenditure 1990-2019. Colombo: Institute for Health Policy 2021.
3. Ministry of Health SL. Annual Health Bulletin, 2019. Colombo: Ministry of Health, 2021.
4. Perera C, Rannan-Eliya RP, Senanayake S, et al. Public Hospital Inpatient Discharge Survey 2005. Colombo: Institute for Health Policy, 2006.
5. United Nations, Department of Economic and Social Affairs, Population Division. World Population Prospects 2022, 2022.
6. Wijemunige , Rannan-Eliya RP, Maurer J, et al. Cost-Effectiveness and Distributional Impact of Opportunistic Screening for People at High-Risk of Cardiovascular Disease in Sri Lanka: A Modelling Study. Glob Heart 2022;17(1):89. doi: 10.5334/gh.1174

Text S2

The WHO-2019 screening tool is accompanied by a Stata program *whocvdrisk* [1] which can calculate 1-year, 5-year and 10-year probabilities of events and deaths for each of CHD, stroke and CVD. For each participant, we calculated 1, 5 and 10-year probabilities of events and deaths for each condition. We fitted quadratic equations to predict the 2, 3, 4, 6, 7, 8, and 9-year probabilities of events and deaths for each participant for each condition, then used the difference between the probabilities of neighbouring years to obtain the probability of each event/death by year (e.g. 10-year probability – 9-year probability = probability of dying in year 9). This method was used so that the full spectrum of data available (1, 5 and 10-year probabilities) could be utilised.

References

1. Emergency Risk Factors Collaboration, University of Cambridge. Cardiovascular Epidemiology Unit: Programs. Available from: https://www.phpc.cam.ac.uk/ceu/erfc/programs/. Accessed 1 July 2023.
